# Monitoring Ti_3_C_2_T_*x*_ MXene Degradation Pathways Using Raman Spectroscopy

**DOI:** 10.1021/acsnano.4c02150

**Published:** 2024-05-06

**Authors:** Sonata Adomaviciute-Grabusove, Anton Popov, Simonas Ramanavicius, Valdas Sablinskas, Kateryna Shevchuk, Oleksiy Gogotsi, Ivan Baginskiy, Yury Gogotsi, Arunas Ramanavicius

**Affiliations:** † Institute of Chemical Physics, Vilnius University, Sauletekio Av. 3, LT-10257 Vilnius, Lithuania; ‡ NanoTechnas—Center of Nanotechnology and Materials Science, Institute of Chemistry, Faculty of Chemistry and Geosciences, Vilnius University, Naugarduko St. 24, LT-03225 Vilnius, Lithuania; § Department of Organic Chemistry, Centre for Physical Sciences and Technology, Sauletekio Av. 3, LT-10257 Vilnius, Lithuania; ∥ A.J. Drexel Nanomaterials Institute and Materials Science & Engineering Department, Drexel University, 3141 Chestnut Street, Philadelphia, Pennsylvania 19104, United States; ⊥ Materials Research Center, Ltd., Krzhyzhanovskogo Str. 3, 03142 Kyiv, Ukraine; # Department of Physical Chemistry, Institute of Chemistry, Faculty of Chemistry and Geosciences, Vilnius University, Naugarduko St. 24, LT-03225 Vilnius, Lithuania; ∇ Department of Nanotechnology, Centre for Physical Sciences and Technology, Sauletekio Av. 3, LT-10257 Vilnius, Lithuania

**Keywords:** 2D materials, MXenes, Raman spectroscopy, TiO_2_ nanoparticles, Ti_3_C_2_T*_x_*, MXene degradation, laser-induced disruption

## Abstract

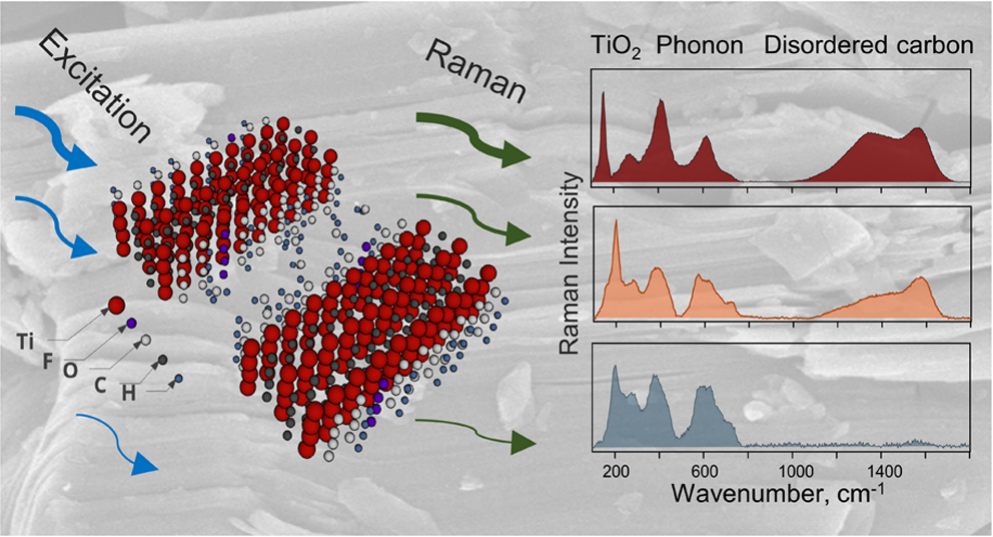

Extending applications of Ti_3_C_2_T_*x*_ MXene in nanocomposites and across fields of electronics, energy storage, energy conversion, and sensor technologies necessitates simple and efficient analytical methods. Raman spectroscopy is a critical tool for assessing MXene composites; however, high laser powers and temperatures can lead to the materials’ deterioration during the analysis. Therefore, an in-depth understanding of MXene photothermal degradation and changes in its oxidation state is required, but no systematic studies have been reported. The primary aim of this study was to investigate the degradation of the MXene lattice through Raman spectroscopic analysis. Distinct spectral markers were related to structural alterations within the Ti_3_C_2_T_*x*_ material after subjecting it to thermal- and laser-induced degradation. During the degradation processes, spectral markers were revealed for several specific steps: a decrease in the number of interlayer water molecules, a decrease in the number of −OH groups, formation of C–C bonds, oxidation of the lattice, and formation of TiO_2_ nanoparticles (first anatase, followed by rutile). By tracking of position shifts and intensity changes for Ti_3_C_2_T_*x*_, the spectral markers that signify the initiation of each step were found. This spectroscopic approach enhances our understanding of the degradation pathways of MXene, and facilitating enhanced and dependable integration of these materials into devices for diverse applications, from energy storage to sensors.

## Introduction

The rapidly expanding family of two-dimensional (2D) materials, MXenes, emerged with the discovery of 2D Ti_3_C_2_T_*x*_ in 2011.^1,2^ MXenes are carbides, nitrides, oxycarbides, and carbonitrides of transition metals with the general formula M_*n*+1_X_*n*_T_*x*_ (where *n* = 1, 2, 3, or 4; M depicts a transition metal, *e.g*., Ti, V, Nb, Mo; X represents C and/or N (O substitution is possible); T_*x*_ refers to surface functional groups, *e.g*., −OH, −F, = O, *etc*.). MXenes are generally produced by selectively etching the middle element A of the parent MAX phase (where A refers to elements from the main groups III–VI, such as Al or Si), thus releasing 2D MX layers. Multiple layers of MXenes are further separated by various intercalants and/or sonication, yielding 2D MXenes. In particular, Ti_3_C_2_T_*x*_ MXene has a long history of research and is notable for inexpensive synthesis from earth-abundant elements and its outstanding properties, such as high conductivity, hydrophilicity, redox-active surfaces, *etc*.^3−5^ Further investigations are also directed toward efficiently producing different MXene types that can yield defect-free 2D MXene layers.^4,6^

The increasing attention to MXene can be attributed, in part, to its wide range of applications across various fields, including energy storage^5,7−9^ and conversion^3,9,10^ (such as supercapacitors and batteries), sensor development,^11−19^ electronic components,^7,8,20−23^ and the fabrication of nanocomposites for numerous other applications.^5^ Many optical,^24^ electronic,^9^ and chemical properties^25^ of MXene depend on structural changes, including variations in material oxidation state,^9,22,24,26^ and the presence of amorphous carbon or TiO_2_ nanoparticles^27−29^ that arise during lattice degradation.^30−33^ The sensitivity of MXene to environmental and structural changes makes it an excellent sensor material. MXene can function as a sensor on its own^11−15^ or be integrated as a crucial component in nanocomposites for sensor applications.^13,16−19^

Raman spectroscopy is a powerful analytical technique used to study the stability and degradation properties of materials,^34−40^ and it is particularly useful for assessing MXene quality during fabrication of devices and sensors. It is sensitive to interactions between chemical species and alterations in lattice structures, making it applicable to detect the presence of amorphous compounds, as well as traces of transition metal oxides commonly found in the structure of MXene. The Raman spectra can reveal detailed information about the structural and surface chemistry of MXenes,^41^ detect TiO_2_ structures,^42−44^ and assess lattice oxidation and degradation,^22,24,45,46^ among other valuable applications.^24,42,43,47^ Raman spectroscopy-guided sensor development provides enhanced performance, while Raman assessment ensures quality during composite fabrication. Moreover, the application of MXene in sensor technology not only benefits from Raman spectroscopy for analysis but also contributes to the advancement of Raman spectroscopy techniques, enhancing their sensitivity and broadening their applicability.^7,8,20,24,48^

The Raman spectral bands of Ti_3_C_2_T_*x*_ MXene have been assigned to lattice phonon modes based on density functional theory calculations and additional computational methods.^41,42,49^ Notably, while most computational predictions were based on homogeneous surface groups (=O, −OH, and −F), MXene terminations appear to be inhomogeneous when synthesized by the wet chemical etching method. The presence of complex surface groups has been confirmed by computational calculations^50−53^ in tandem with analytical techniques,^54,55^*e.g*., pair distribution function (PDF), X-ray photoelectron spectroscopy (XPS), electron energy loss spectroscopy (EELS), and Raman spectroscopy.^24,43,49,51,55^ Due to the mixed terminations and defects in the MXene lattice, the deconvolution of Raman spectra can be very complicated. Nevertheless, a recent study utilized machine-learning force field computations to provide further insights into the nature of complex Raman bands by incorporating computational Raman modes for hybrid surface groups along with the concept of symmetry breaking in the lattice.^51^ This approach successfully explained the broadening of Raman bands and the appearance of additional in-plane modes when considering hybrid −O(OH) surface groups.

The primary objective of this study is to uncover the spectral changes observed in treated MXene films. It includes tentative assignments of these features to the vibrational modes discussed in the literature.^24,43,49,51^ However, it should be emphasized that the observed changes in the Raman spectra are not exclusive to any specific termination type. Instead, they are a result of the overall structural complexity arising from the presence of various terminations in the MXene samples. This study also highlights the effects of film degradation on MXene Raman spectra, which encompass oxygenation and deterioration of the MXene lattice, reduction of interlayer water, and other changes.^9,56−59^ In particular, we focused on the Raman spectral markers associated with processes resulting from laser-induced changes, such as oxidation and partial destruction of the lattice. Further investigations of lattice transitions were conducted by using the thermal treatment of MXene films. In general, spectral differences in the Raman spectra of the degraded MXene films were found, which can be attributed to (I) a decrease in the number of molecules trapped between the MXene sheets, (II) the decrease in the number of −OH groups, and (III) oxidation of MXene. Understanding MXene degradation pathways may contribute to the design of energy storage and sensor materials with improved stability. Therefore, the findings presented in this study guide sensor development, enabling precise detection in diverse applications.

## Results

### Scanning Electron Microscopy (SEM), Energy-Dispersive X-ray (EDX) Analysis, X-ray Diffraction (XRD), and Visible–Near-Infrared (Vis–NIR) Spectroscopy Characterization of Synthesized MXene

**Figure 1:**
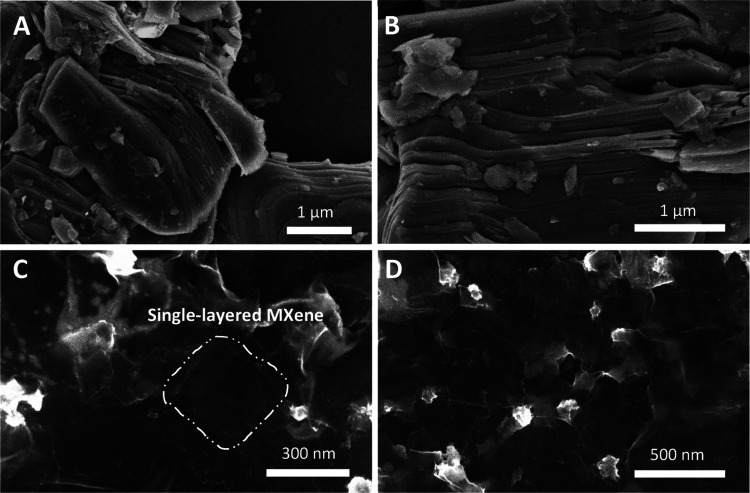
SEM images of multilayered powder (A, B) and delaminated single-layered films of (C, D) Ti_3_C_2_T_*x*_ MXene.

The morphology of synthesized MXene was investigated with SEM imaging (Figure 1). The stacked lamellae were observed in the Ti_3_C_2_T_*x*_ multilayered MXene (Figure 1A,1B). After delamination, single-layered MXene flakes were formed and are shown in Figure 1C,D.

**Figure 2:**
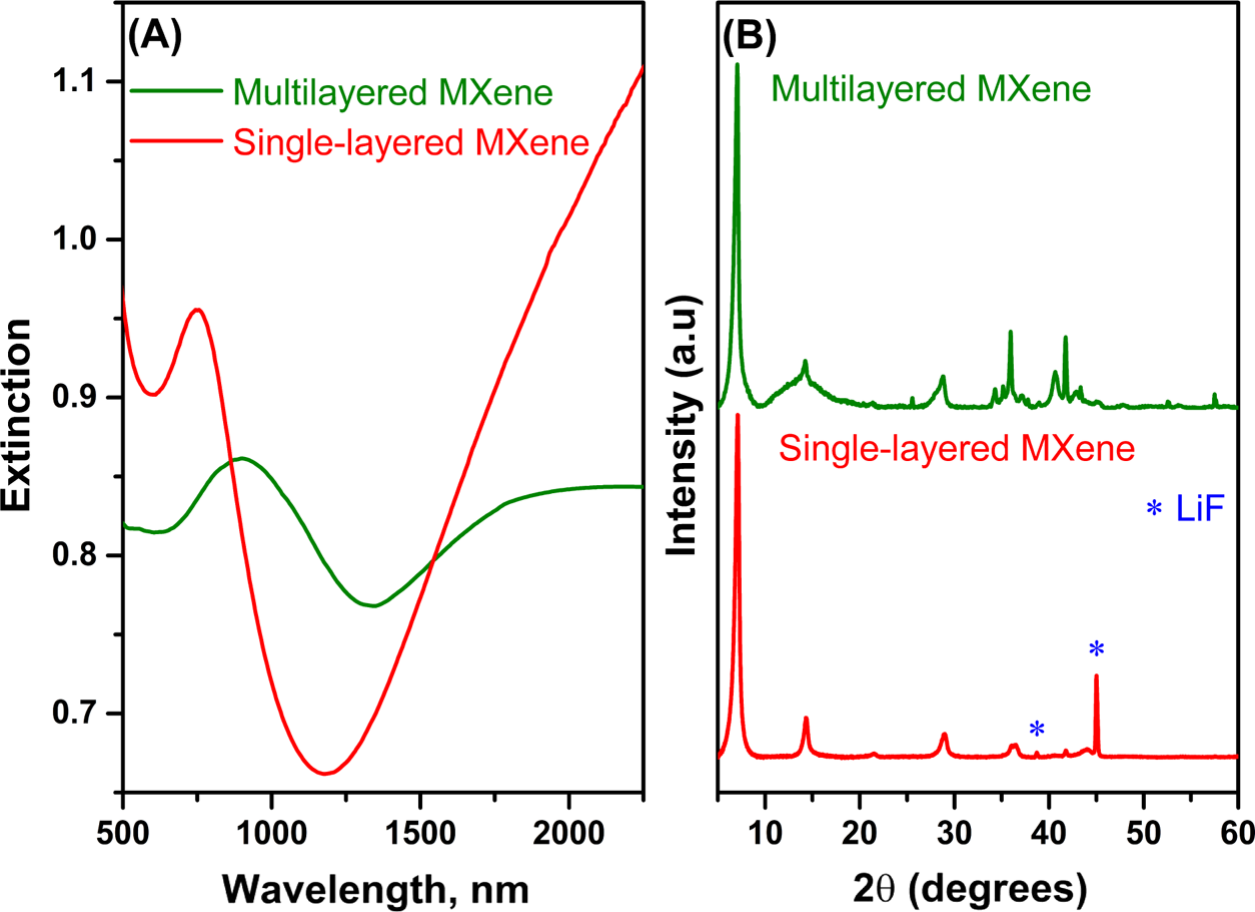
Comparison between the multilayered and single-layered MXene samples. (A) Vis–NIR absorption spectrum and (B) XRD patterns of multilayered and delaminated single-layered MXene.

Delamination efficiency was confirmed by EDX analysis (Figure S1), which showed the presence of traces of aluminum, suggesting successful etching of the MAX phase and the formation of multilayered MXene. The aluminum residues were present as relatively uniform amorphous structures on the surface of the MXene sheets and did not affect the parameters investigated in this study. XRD analysis also confirmed the successful etching and delamination of the MXene samples (Figure 2B). The XRD patterns of synthesized MXene are presented in Figure 2B. The results clearly show that the MXene was synthesized, as indicated by the shift of the (002) peak to 7.0 and 7.1° in the case of multilayered and single-layered MXene, respectively.^60^ However, the XRD pattern of multilayered MXene (Figure 2B) from 33 to 44° is similar to that of the MAX phase,^11^ suggesting incomplete etching and/or washing. In the case of single-layered MXene, two additional diffraction peaks at 38.7 and 45° are observed, which match the XRD data of LiF (00-004-0857) and indicate the presence of residual LiF after washing the MXene samples.

The differences between the multilayered and single-layered samples are also visible in the vis–NIR extinction spectra (Figure 2A). The spectra match the literature.^48^

The extinction spectrum of MXene, obtained after the delamination step, reveals an extinction band at ≈750 nm originating from interband transition.^20,48,61−63^ Additionally, the extinction observed at wavelengths above 1100 nm has been previously assigned to plasmonic oscillations.^48,61^ Our previous research reported increased energy losses, resulting from electronic transitions in the near-infrared (NIR) range for Ti_3_C_2_T_*x*_ MXene.^11^ Generally, the overlap between the plasmonic frequencies and electronic transitions leads to less pronounced and wide-band plasmon resonances,^64,65^ which are observed in our case. The multilayered MXene is expected to have lower extinction in this range due to its lower charge-carrier density than single-layered MXene and different surface functional groups.

To conduct a comprehensive qualitative study of MXene, dried MXene films were separately stored in oxygen and nitrogen gas environments and investigated using both vis–NIR and Raman spectroscopy (Figures S2 and S3). The observed spectral differences between the freshly formed films and those stored in oxygen may be attributed to the partial oxidation of MXene. Previous studies have shown that the extent of oxidation depends on the oxygen content during storage^47,66,67^ and temperature.^27^

### Raman Spectroscopy Characterization of Single-Layered and Multilayered MXene

A further distinction between the single-layered and multilayered Ti_3_C_2_T_*x*_ MXene can be made by vibrational spectroscopy, *e.g*., by investigating Raman scattering. Generally, two spectral regions are important for Raman spectroscopic analysis of MXene: the low-frequency (100–800 cm^–1^) one, which represents lattice vibrations (phonons), and the region from 1000 to 1800 cm^–1^, which represents C–C stretching vibrations of carbon structures occurring in MXene films.

**Figure 3:**
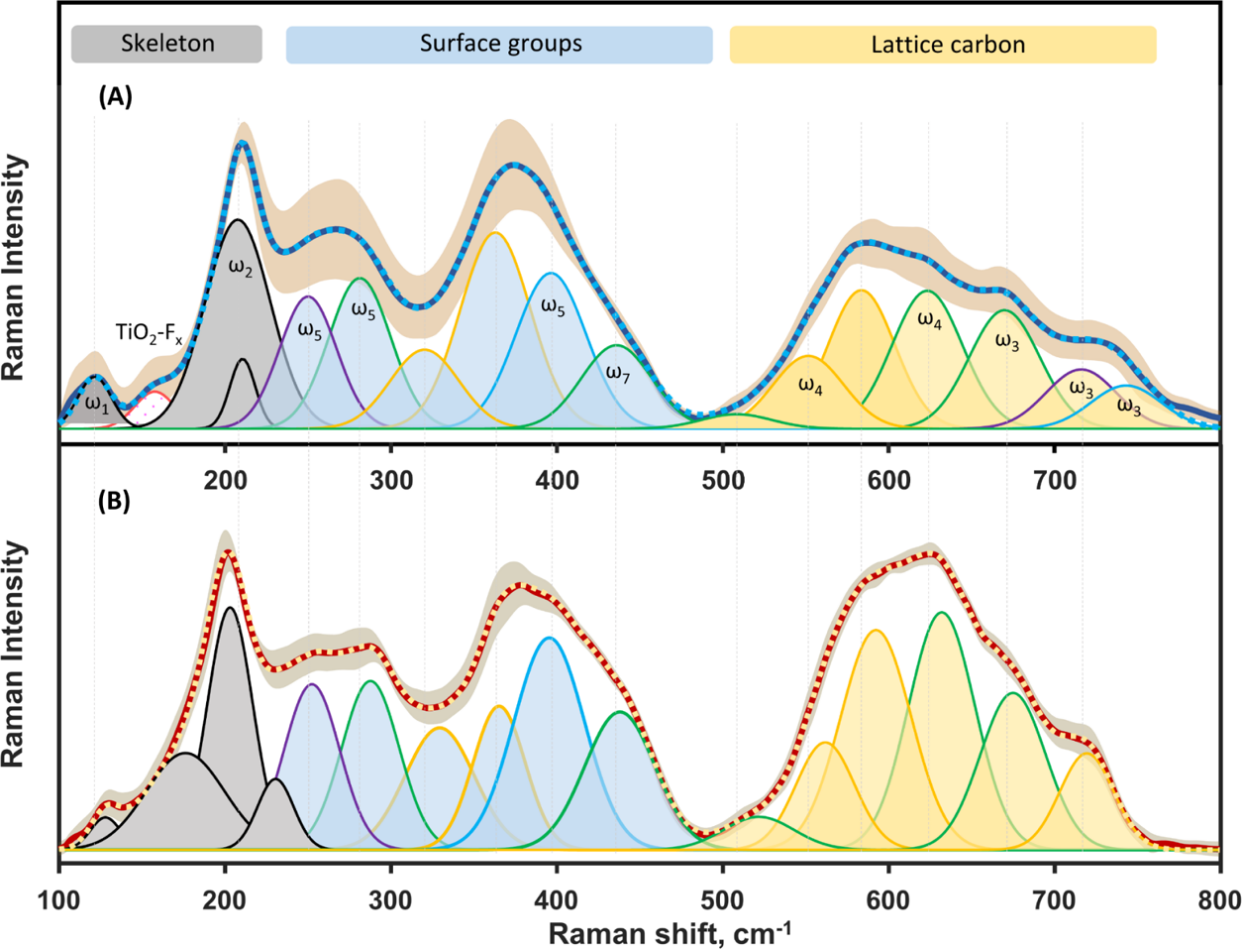
Deconvoluted Raman spectra in low-frequency (100–800 cm^–1^) spectral region of multilayered (A) and single-layered Ti_3_C_2_T_*x*_ MXene (B). The excitation wavelength was 633 nm. Spectra are separated into spectral regions: skeleton vibrations of all lattice atoms (gray); the surface group region that mainly consists of vibrations associated with the outermost atoms in the material (blue); the lattice carbon region that represents phonon modes of the carbon atoms (yellow). The peak profiles mark our proposed assignment of phonon modes from different homogeneously terminated Ti_3_C_2_T_*x*_. Modes associated with Ti_3_C_2_(OH)_2_ are outlined with the green line, Ti_3_C_2_O_2_ with the blue line, Ti_3_C_2_F_2_ with the violet line, and the complex peak profiles, constituting several MXene types, with the yellow line. The peak profile arising from Ti oxyfluoride is outlined with a pink line.

In the Ti_3_C_2_T_*x*_ fingerprint region, the computational approach was able to provide mode assignments for homogeneously terminated MXene and explain the appearance of additional in-plane modes in MXene with heterogeneous terminations—two at approximately 300–400 cm^–1^ and two at 550–650 cm^–1^. Our research incorporated these heterogeneous modes in the deconvoluted Raman profile of single-layered and multilayered MXene (Figure 3). Despite the difficulty in interpreting the Raman bands, the fitted Gaussian peak profiles align well with the proposed mode positions in this spectral region. Additionally, previous works suggested several markers for increasing =O content in MXene, such as blue shifts of the bands at approximately 700 and 395 cm^–1^ and a red shift of the band at approximately 620 cm^–1^.^51^ Correspondingly, in the case of voltage-induced oxidation of MXene, a red shift of the 620 cm^–1^ band and blue shift of the 395 cm^–1^ band were observed as well.^22,24,43,45^

Generally, Raman spectra of single-layered and multilayered MXene possess only minor differences. These differences might arise from various factors, including the number of synthesis steps,^42,43,47^ variations in the interlayer spacing, and the presence of water and potential intercalants between the layers (Figure 3). The Raman spectra of multilayered MXene exhibit comparably lower intensity bands in the 560–800 cm^–1^ spectral region, which are mainly associated with the vibrations of the lattice carbon atoms. A notable reduction in intensity is observed for the complex band at approximately 620 cm^–1^, which we mainly attribute to the in-plane vibrations of the carbon atoms. The complexity arises from different ratios of =O, −OH, and −F terminations affecting this and most other bands in MXene spectra. Several studies have shown that the red shift of this band indicates the oxidation of MXene.^22,45,51^ Thus, in this research, we assigned the spectral region at approximately 590 cm^–1^ to Ti_3_C_2_O_2_ MXene structures, while the region at approximately 667 cm^–1^ was assigned to Ti_3_C_2_(OH)_2_ MXene structures (Table 1 and Figure 3).

**Table 1:** Experimentally Observed Raman Bands of Single-Layered and Multilayered Ti_3_C_2_T_*x*_ MXene^a^

vibrational frequency, cm^–1^				
single-layered		multilayered		
633 nm	785 nm	633 nm	785 nm	MXene type^49,51^
122	122^b^	122	123^b^	complex^49^
154	154	154	154	
200	201	211	210	complex^49^
256	258	258^c^	258	Ti_3_C_2_F_2_^49,51^
283	283	284	283	Ti_3_C_2_(OH)_2_^49^
372	372^b^	370	371^b^	complex^51^
450		452		Ti_3_C_2_(OH)_2_^49^
511	513^b^	505^c^	512^b^	Ti_3_C_2_(OH)_2_^49^
590	584	590	585	Ti_3_C_2_O_2_^49,51^
626	617	621	616	complex^51^
667	667	667	668	Ti_3_C_2_(OH)_2_^49,51^
712	722^b^	734	737^b^	Ti_3_C_2_O_2_^49^
1396	1396			
1582	1582	1562	1561	

a Complex—two or more different MXene types.

b Increased intensity in spectral band compared to 633 nm excitation.

c Not prominent or weak band.

The most prominent contrast between the single-layered and multilayered MXene becomes evident in the band associated with the ω_2_ mode (out-of-plane skeleton vibration of all atoms in the lattice, Table S1). The loosening of the ω_2_ mode due to less confined out-of-plane vibrations in single-layered MXene leads to an observed red shift of the band from 211 to 200 cm^–1^ (Table 1). Similarly, a red shift is observed for the other out-of-plane vibrational modes at 734 cm^–1^ in multilayered MXene to 711 cm^–1^ in single-layered MXene. Notably, the latter band also shows sensitivity for the −O or −F content on the surface^51^ and further underscores its dependence on the synthesis route.^42^

When comparing the Raman spectra of single-layered MXene collected using different excitation wavelengths, notable changes are observed primarily under preresonance conditions (Figure S4). From vis–NIR extinction spectra, the resonance Raman condition for MXene samples in our study was excitation with a wavelength of 750 nm (Figure 2). Excitation with a 785 nm laser provides photon energy close to the resonance Raman condition. The preresonance excitation yields Raman spectra with high intensity of a few resonant spectral bands (Figure S5). These bands include 122 cm^–1^ (“skeleton vibration”), which is associated with the in-plane vibrational mode of all atomic groups, 513 cm^–1^ (associated with the out-of-plane ω_6_ mode of Ti_3_C_2_(OH)_2_), and 722 cm^–1^ (associated with the out-of-plane ω_3_ mode of mainly Ti_3_C_2_O_2_) bands.

### Laser-Induced Disruption of MXene

**Figure 4:**
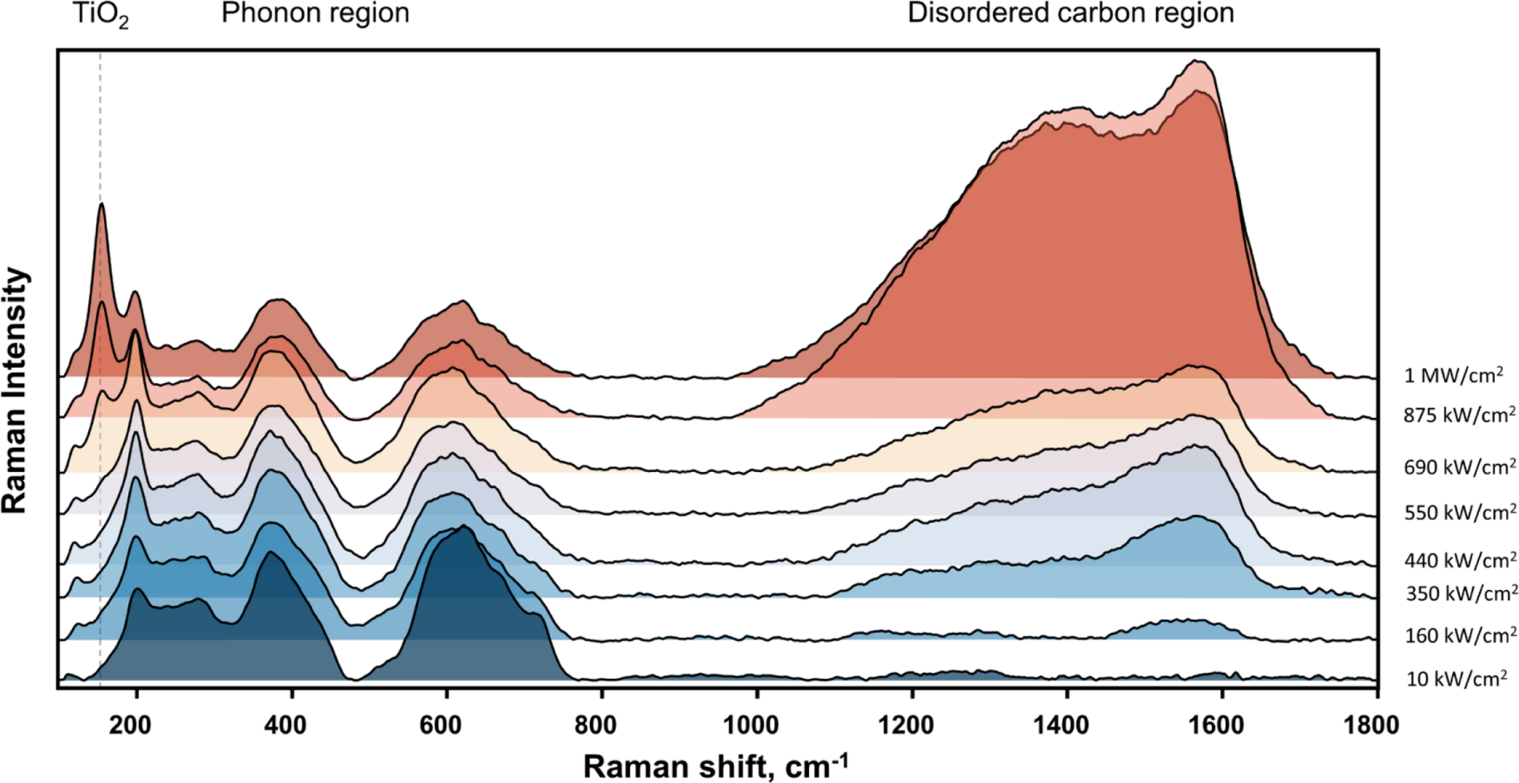
Changes in the Raman spectrum of Ti_3_C_2_T_*x*_ are due to laser radiation. The laser power density varied from 10 kW/cm^2^ to 1 MW/cm^2^. Three spectral regions are marked as TiO_2_ (spectral region of titanium oxide formation), phonon region (lower frequency vibrations), and disordered carbon region (vibrations of C–C bonds). The applied excitation wavelength was 633 nm.

The disruption of the MXene lattice was achieved by using different laser wavelengths: 633, 532, and 457 nm. Each laser generates a distinct power density on the sample and induces lattice defects, which can be traced by Raman spectroscopy. The evolution of phonon bands was first examined using a 633 nm laser in the power density range of 10 kW/cm^2^–1 MW/cm^2^ (Figure 4). Additionally, samples were analyzed at a higher laser power density, which enables a rapid deterioration of the lattice, using a 532 nm laser (with the power density range of 20 kW/cm^2^–26 MW/cm^2^) and a 457 nm laser (with the power density range of 240 kW/cm^2^–4.9 MW/cm^2^) (Figures S7–S10). Comparable spectral patterns were observed for various excitation wavelengths at equivalent power densities. Reasons for MXene deterioration were previously identified and include (i) inner titanium (Ti) atom diffusion to outer layers in the presence of lattice defect; (ii) the formation of C–C bonds between different MXene planes; and (iii) the formation of TiO_2_.^68^

The first apparent sign of lattice disruption in MXene can be seen in the disordered carbon spectral region of 1000–1800 cm^–1^ (Figure 4). Amorphous carbon and hydrocarbons begin to form at excitation powers of 160 kW/cm^2^. Broad G and D bands can be observed at 1582 cm^–1^ (full width at half-maximum—fwhm of 300 cm^–1^) and 1396 cm^–1^ (fwhm—120 cm^–1^), respectively. The G band appears from C–C bond vibration in all sp^2^ hybridized carbon systems, while the D band appears in disordered carbon ring systems.^69,70^ Under 633 nm excitation, the intensity of the G band of disordered carbon continuously increased with increasing power density, reaching its maximum at 875 kW/cm^2^. As the laser power density is further increased, the disordered carbon bands gradually weaken due to the widespread deterioration of C–C bonds, as shown in Figure 4.

The final and most abrupt step of MXene deterioration, namely, the formation of TiO_2_ nanoparticles, was observed at a power density of 550 kW/cm^2^ with 633 nm excitation. The formation was observed at lower power densities: 330 and 390 kW/cm^2^ with 532 and 457 nm excitations, respectively, and advancing until approximately 1.5–1.6 MW/cm^2^ (Figures S7–S10). TiO_2_ formed from MXene can exist in two crystal structures: anatase and rutile. The anatase phase is the first to appear as it requires less energy to form. The anatase phase was identified by the most intense band at 154 cm^–1^ associated with the E_g_ mode. In our work, this mode appeared blue-shifted at low laser power densities, which is a sign of defects and oxygen deficiency in the anatase structure.^71−73^ A higher content of anatase TiO_2_ in the MXene sample is indicated by additional markers–spectral bands at 406 and 633 cm^–1^, which are associated with B_1g_ and E_g_ modes, respectively, along with the red shift of E_g_ mode to 144 cm^–1^. Further increasing the laser power density initiated the formation of the rutile phase of TiO_2_. Spectral markers of the rutile phase start to dominate in the Raman spectra when the laser power density exceeds 2 MW/cm^2^ (Figures S8–S10).

### Low-Frequency Raman Spectrum of MXene Samples Subjected to Laser-Induced Disruption

**Figure 5:**
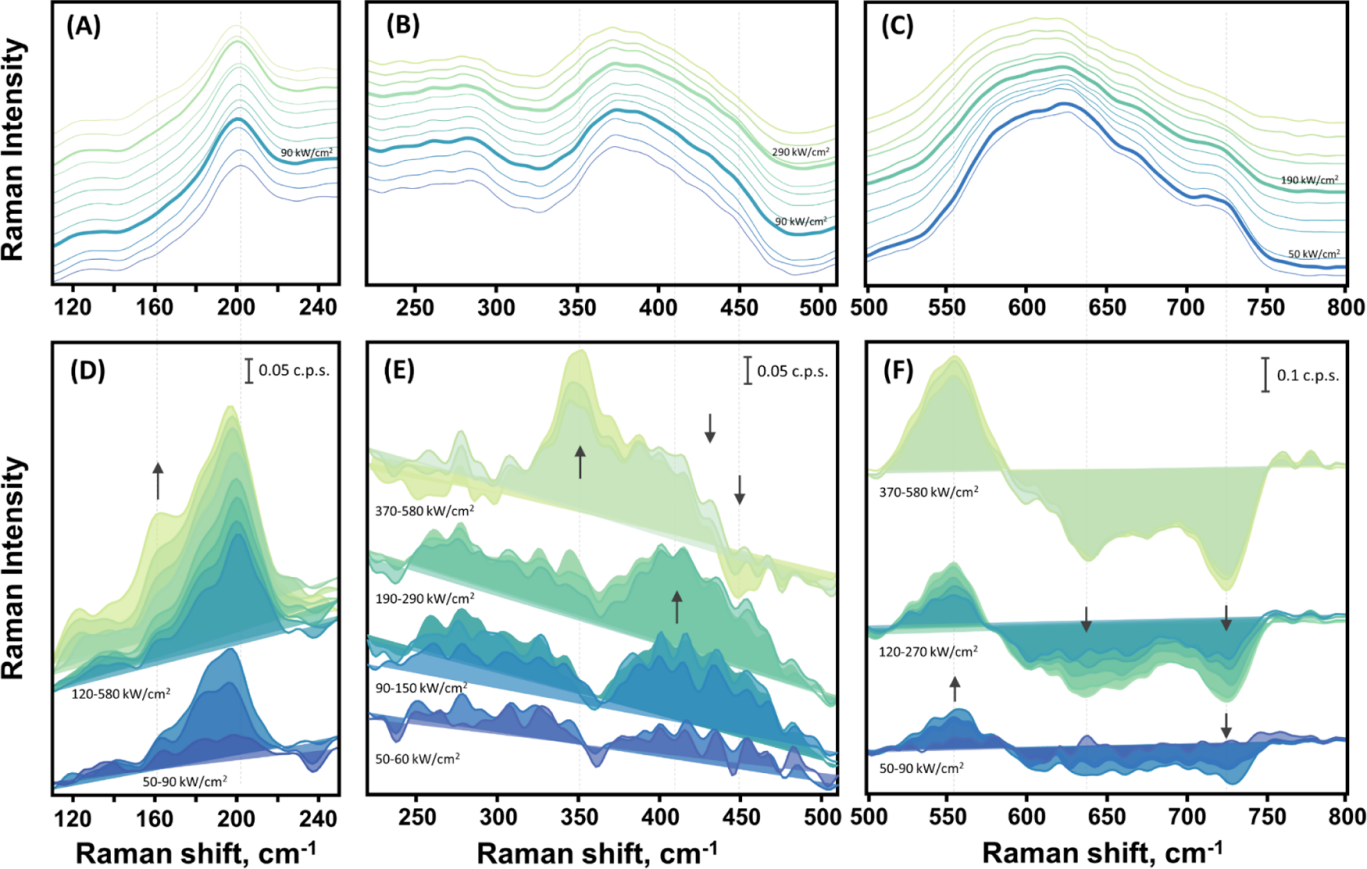
Low-frequency (100–800 cm^–1^) Raman spectrum of deteriorating Ti_3_C_2_T_*x*_ MXene due to 633 nm laser illumination (A–C) along with the corresponding differential spectra (D–F). The laser power density was changed: from 30, 50, 60; 90, 120, 150, 190, 230, 290, 370; and 470 to 580 kW/cm^2^ with the spectra arranged from bottom to top in that order. To enhance clarity, the differential spectra, illustrating newly emerging spectral markers, are presented separately in the bottom panels. The arrows indicate changes in the spectra that emerge with an increasing laser power density. Data were smoothed with a Savitzky–Golay filter, and the unsmoothed spectra are shown in Figure S6.

Spectral changes of photodisrupted MXene are also observable in the phonon region of 100–800 cm^–1^ (Figure 5A–5C). Lattice vibrations were analyzed using laser excitation at a power density range of 30 to 580 kW/cm^2^. More intense laser light severely disrupts the MXene layers. Therefore, the results for such samples are not included in the analysis. No significant differences were found when using various wavelengths for the excitation of Raman scattering (Figure S4). Consequently, we focus on the results obtained with 633 nm excitation (Figure 5).

The analysis of lattice phonon bands was conducted through differential spectra (Figure 5D–F). Initially, the spectrum obtained using the lowest laser power density was subtracted from the presented spectra. As the laser power density increased to 60 kW/cm^2^, the 199 cm^–1^ spectral band, associated with the ω_2_ mode, and the surface group bands increased in intensity, while the lattice carbon region became less intense. A slight red shift was observed in the 199 cm^–1^ band at 60 kW/cm^2^, likely due to the heating of the sample. The upturn in laser power density to 90 kW/cm^2^ revealed a pronounced blue shift for the ω_2_ mode to 204 cm^–1^ (Figure 5A,D). Indeed, the blue shift is not expected and indicates the stiffening of the mode due to the removal of water molecules trapped between the MXene layers.^57,59^ It has been shown that the loss of water and surface functional groups reduces the interlayer spacing, causing individual monolayers to converge,^9,56,58,59^ though the loss of surface groups requires a higher temperature to desorb.^59^

In our study, we observed changes linked to increasing =O content in MXene^22,24,43,45^ in addition to the reduction of interlayered water. Specifically, a blue shift in the complex band at 372 cm^–1^ was noted (Figure 5C). This blue shift was observed when the excitation power density was between 90 and 290 kW/cm^2^, before anatase formation, indicating oxidation of the MXene.^24,42,43^ The spectral region at 410 cm^–1^ displayed increased intensity within this power density range, which contributed to the observed blue shift in the 372 cm^–1^ band (Figure 5E). Another indication of oxidation is the red shift of the wide complex band at 626 cm^–1^.^24^ This red shift, peaking at 620 cm^–1^, is noticeable in the spectra starting from 190 kW/cm^2^ (Figure 5C). However, differential spectra reveal that changes in this area appeared much sooner. Furthermore, we observed the gradual increase of the band at 550 cm^–1^, which is associated with Ti_3_C_2_O_2_ structures in this work, together with a decrease of the band at 640 cm^–1^, which we link to Ti_3_C_2_(OH)_2_. These changes were observed even at a low laser power density of 50 kW/cm^2^ and persisted as the power density reached 580 kW/cm^2^ (Figure 5F). The decrease in the spectral region at 640 cm^–1^ became more pronounced at 190 kW/cm^2^. Interestingly, we did not observe a blue shift of the band at 372 cm^–1^ when anatase was formed (when the sample was excited by 370 kW/cm^2^ power density). However, we did observe a red shift of the complex band at 620 cm^–1^ in this case.

The bands at 283 and 256 cm^–1^ experience a rapid decrease starting at 370 kW/cm^2^ (Figure 5B). The spectral band of anatase TiO_2_ at 154 cm^–1^ appears together with the rapid growth of the band observed at 350 cm^–1^ when the sample is excited by a power density of 370 kW/cm^2^. Other studies have reported that the band at 350 cm^–1^ is associated with the presence of −F the MXene.^43^ However, our results suggest that the band at 350 cm^–1^ might be associated with the formation of TiO_2_ structures, since they appear together.

Analyzing the intensity ratios of Raman bands associated with various vibrational modes of MXenes with different terminations can provide valuable insights into structural changes. This approach allows us to clearly distinguish indications of interlayer water reduction, MXene oxidation, and lattice disruptions. However, as previously mentioned, most of the MXene bands are complex; therefore, the assignments for these bands should be made with caution. This study focuses on analyzing the intensity changes of the most prominent MXene Raman bands. To enhance readability, we made tentative assignments of these bands to the lattice modes of Ti_3_C_2_O_2_ and Ti_3_C_2_(OH)_2_.

Specifically, we calculated the intensity ratios of the ω_2_ mode and the peaks at 372 or 283 cm^–1^, which were related to the ω_5_ modes of either =O-terminated MXene (*I*_ω2_/*I*_ω5(O)_) or −OH-terminated MXene (*I*_ω2_/*I*_ω5(OH)_), respectively. Given the complexity of the bands, we also considered other ratios between the ω_2_ mode and peaks at 590 or 626 cm^–1^, assigned to the ω_4_ modes of = O-terminated MXene (*I*_ω2_/*I*_ω4(O)_), or −OH-terminated MXene *I*_ω2_/*I*_ω4(OH)_ as complementary information. These intensity ratios offered insights into the changes in surface groups.

**Figure 6:**
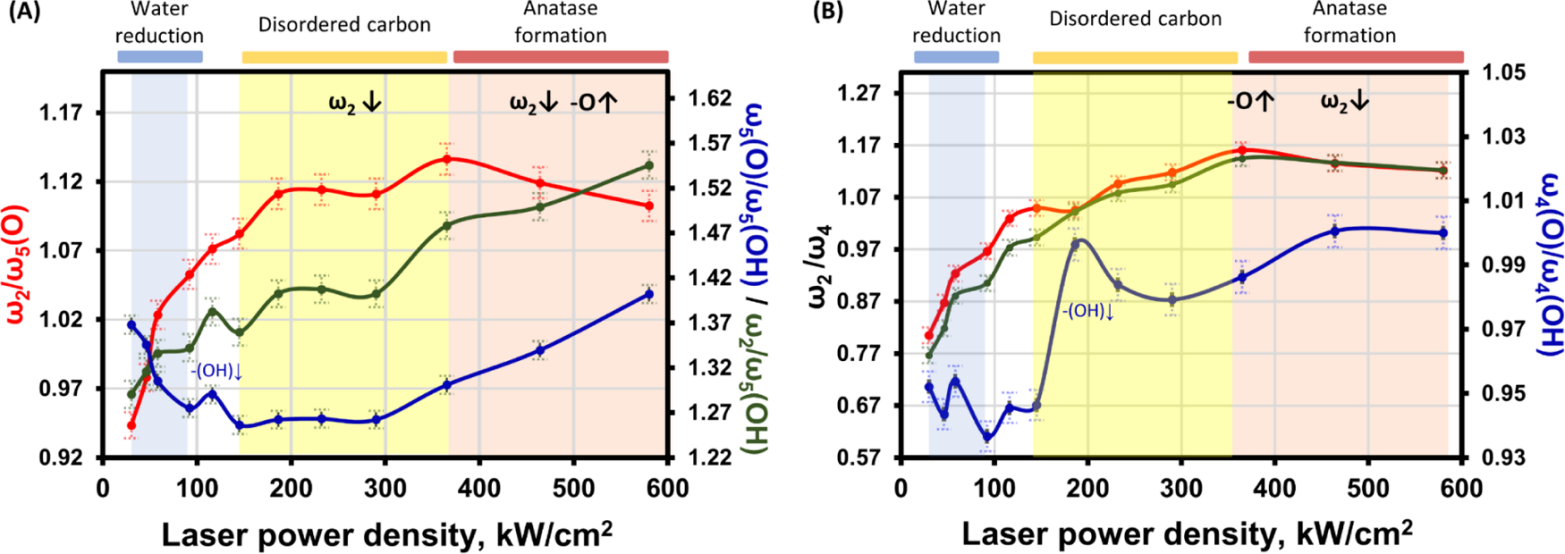
Intensity ratios of MXene Raman bands associated with different vibrational modes of Ti_3_C_2_O_2_ and Ti_3_C_2_(OH)_2_. The intensity ratios *I*_ω2_/*I*_ω5(O)_ (red line), *I*_ω2_/*I*_ω5(OH)_ (green line), and *I*_ω5(O)_/*I*_ω5(OH)_ (blue line) of ω_2_ and ω_5_ modes of either = O or −OH-terminated MXene (A). The intensity ratios *I*_ω2_/*I*_4(O)_ (red line), *I*_ω2_/*I*_ω4(OH)_ (green line), and *I*_ω4(O)_/*I*_ω4(OH)_ (blue line) of ω_2_ and ω_4_ modes of either =O or −OH-terminated MXene (B). The main changes in the MXene lattice are noted above the graph: –O↑—increasing oxidation; ω_2_↓—reduction of the ω_2_ mode intensity. The main processes distinguished by spectral markers appearing in the spectra are noted above the graphs: reduction of interlayered water, formation of disordered carbon bands, and formation of anatase.

In general, the intensity of the ω_2_ mode increases with an increasing power density at low levels. The *I*_ω5(O)_/*I*_ω5(OH)_ ratio, on the other hand, decreased from 1.37 to 1.26 (at 150 kW/cm^2^) and then began increasing at 370 kW/cm^2^, reaching 2.1 (at 1 MW/cm^2^) (Figure 6A, blue curve). The decrease in the *I*_ω5(O)_/*I*_ω5(OH)_ ratios at low laser power densities could be explained by the changes in interlayer spacing from the water in the sample. This correlates to the blue shift of the ω_2_ mode at 90 kW/cm^2^, indicating the water reduction. After the complete water reduction (at 120 kW/cm^2^ laser power density), the amount of –OH surface groups decreases, as was observed in annealing studies.^57^ It is marked by a bump in the green and blue curves in Figure 6A at 120 kW/cm^2^ together with a rapid increase in the *I*_ω4(O)_/*I*_ω4(OH)_ ratio from 0.95 to 1.00 at 190 kW/cm^2^ (Figure 6B, blue curve).

Following this process, lattice deterioration becomes evident from 150 kW/cm^2^ by the onset of C–C formation, as indicated by the appearance of spectral bands related to disordered carbon (Figure 4). This deterioration is characterized by a decrease in the *I*_ω2/_*I*_ω5(OH)_ and *I*_ω5(O)_/*I*_ω5(OH)_ ratios. Notably, the Ti_3_C_2_(OH)_2_ MXene is supposed to be the least stable.^57^ Another sign of Ti_3_C_2_(OH)_2_ diminishing is evidenced by the sharp increase in the *I*_ω4(O)_/*I*_ω4(OH)_ at 190 kW/cm^2^. The decrease in *I*_ω4(O)_/*I*_ω4(OH)_ ratio (Figure 6B, blue curve) is observed for the range 190–290 kW/cm^2^, which coincides with the increasing intensity of disordered carbon bands and indicates further lattice deterioration.

The sudden rise in *I*_ω2_/*I*_ω5(O)_, *I*_ω2_/*I*_ω5(OH)_, together with a rise in *I*_ω5(O)_/*I*_ω5(OH)_ and *I*_ω4(O)_/*I*_ω4(OH)_ ratios at 370 kW/cm^2^ indicates the initiation of anatase formation and further oxidation of the Ti_3_C_2_T_*x*_ MXene, presumably signifying the diminishing of surface groups. As oxidation progresses (with increasing laser power density), the most noticeable change is the decreasing *I*_ω2_/*I*_ω5(O)_ ratio. Interestingly, the marker for lattice degradation just before the appearance of the anatase band (characterized by a bump at 154 cm^–1^ in the spectra) can be observed from these ratios, as well. The *I*_ω2_/*I*_ω5(O)_ and *I*_ω2_/*I*_ω5(OH)_ ratios show a slight decrease just before the appearance of the anatase band in the spectra (at 290 kW/cm^2^).

### Impact of Annealing Temperature on MXene Samples

Temperature-induced changes in the MXene lattice occur at elevated temperatures. As previously mentioned, the thermal effect alters both the surface functional groups and the lattice structure of the MXene while reducing the number of water molecules trapped between the monolayers.^9,56−58,74^ To distinguish between reversible and irreversible lattice changes, MXene films were dried on a SiO_2_-based substrate and then annealed at various temperatures from 50 to 500 °C. The efficiency of these effects can be investigated using the Raman spectra of annealed MXene samples. To accomplish this, Raman spectra were obtained with preresonance (785 nm) and nonresonance (633 nm) excitations before and after annealing.

**Figure 7:**
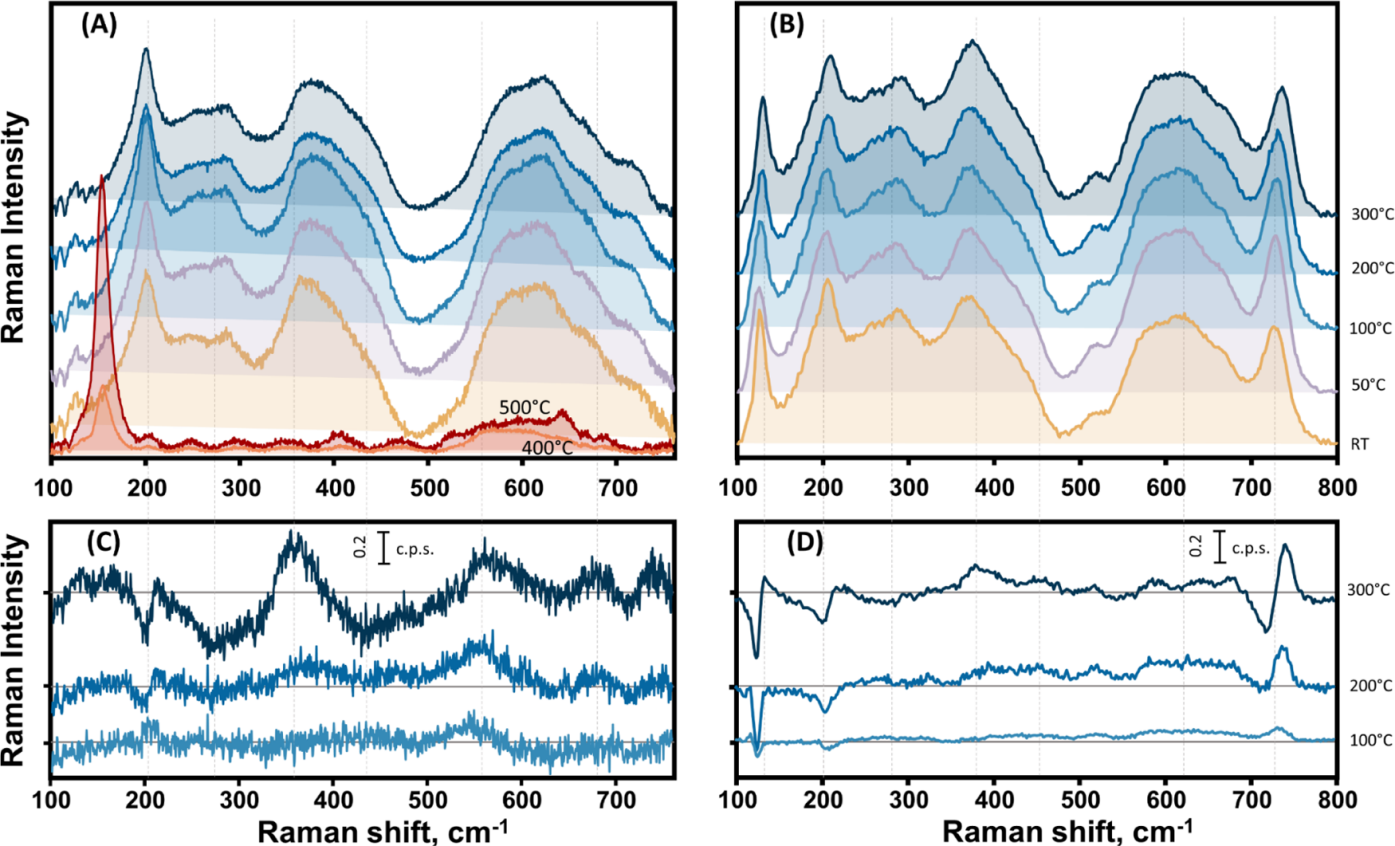
Raman spectra in the phonon spectral region (100–800 cm^–1^) of Ti_3_C_2_T_*x*_-based MXene deteriorating due to heating. Raman excitation wavelengths were 633 nm (A) and 785 nm (B). Subtracted spectra are presented in (C, D) at excitation wavelengths of 633 and 785 nm, respectively. MXene treatment temperatures of 50, 100, 200, and 300 °C are indicated at the right axis of the figure. Spectra recorded after heating to 400 and 500 °C are shown in (A).

Almost no difference was observed when comparing the Raman spectra of the sample before and after heating to 50 °C (Figure 7A,7C). However, a significant increase in the intensities of the wide G and D bands in the carbon spectral region at 1582 and 1396 cm^–1^ is observed as the temperature increases from 200 to 300 °C (Figure S11). When the MXene sample is heated to 400 °C, the intensities of the carbon spectral bands decrease and a new band belonging to the defective TiO_2_ anatase phase appears at 154 cm^–1^. At 500 °C, no carbon bands are detected, and only anatase bands at 154, 406, and 633 cm^–1^ are observed (Figure 7A).

In the phonon spectral region of the MXene sample, almost no spectral changes are observed after heating at 50 °C, except for a slight increase in intensity of the 201 cm^–1^ band, which may indicate the initiation of reduction of interlayered water, as discussed earlier.^56^ The shifts in phonon bands, regarded as spectral markers for a reduction in interlayered molecules, were not discernible for 633 nm excitation (Figure 7C). Heating to 300 °C resulted in red shifts of surface termination bands from 372 to 368 cm^–1^ (associated with MXene oxidation) and from 256 to 246 cm^–1^. The latter shift correlates with an intensity drop at 283 cm^–1^ (Figure 7C). We related this red shift to the decrease in −(OH) surface groups due to several factors: Ti_3_C_2_(OH)_2_ is supposed to be less stable and degrade first,^57^ and the computational studies of MXene indicate the presence of Ti_3_C_2_F_2_ vibrational mode in this spectral range with generally stable mode position.^49,51^ It is worth mentioning that contrary to the laser-induced study, no blue shift of the 372 cm^–1^ band or signal increase at 410 or 450 cm^–1^ was observed during annealing.

The excitation at 785 nm provides complementary results (Figure 7B,7D). Annealing at 50 and 200 °C resulted in a coincident blue shift of the ω_2_ mode from 201 to 208 cm^–1^, respectively. Noticeable changes compared with excitation at 633 nm were observed for the resonant bands at 722 and 122 cm^–1^ (Table 1). These bands show gradual blue shifts from 722 to 734 cm^–1^ and from 122 to 127 cm^–1^ as the temperature increases from 50 to 300 °C. The blue shift of the bands at 122 and 202 cm^–1^ may indicate the removal of interlayer water.

Based on the results of the laser-induced study, several irreversible changes in the MXene lattice were observed. First, a reduction in interlayered water was observed from 200 °C, as indicated by the blue shift of the ω_2_ mode. Second, the band at approximately 626 cm^–1^ was red-shifted to 550 cm^–1^, indicating oxidation of the MXene, starting from 100 °C. Third, further oxidation was indicated by a decrease in intensity of the band at approximately 280 cm^–1^, accompanied by an increase in intensity of the band at 372 cm^–1^ (for MXene heated at 200 °C). Fourthly, progressing oxidation was indicated by a significant decrease in the bands at 283 cm^–1^, at ∼435 cm^–1^, and at 626 cm^–1^ (associated with the Ti_3_C_2_(OH)_2_ MXene) and an increase in intensity at 550 cm^–1^ (associated with the oxidation of MXene structure). Lastly, a new band at 154 cm^–1^ was observed for MXene heated at 400 °C, indicating the formation of anatase TiO_2_.

## Discussion

While we observed significant spectral changes following MXene film treatments (both laser-induced and heat-induced degradation), interpretation of these changes is challenging due to the lack of computational validation for heterogeneous surface terminations. However, these spectral changes are consistent with the results of other annealing and MXene treatment studies.^22,45,51,57^

The spectral changes during the MXene film treatment with a laser were observed and can be outlined in several steps. (I) The reduction of interlayered molecules (together with water) was observed from the blue shift of the band at 200 cm^–1^ at low power densities. (II) A further increase in power density resulted in reduction in surface groups. We hypothesize that this reduction can be inferred from the blue shift of the 370 cm^–1^ band coupled with a spike in the *I*_ω2_/*I*_ω4(OH)_ ratio, indicating a decrease in −(OH) surface groups.^49^ Another indication of oxidation, the red shift of the 620 cm^–1^ band, was noticed at higher power densities but was visible even during the formation of TiO_2_ structures. These spectral markers indicate changes in surface groups, allowing us to pinpoint when these changes initiate. The blue shift of the 370 cm^–1^ band is observed first, though it only occurs at lower power densities. Differential spectra unveil subtler changes occurring even at lower laser power densities for the 620 cm^–1^ band. We observed a gradual increase in the band at 550 cm^–1^, which was accompanied by a decrease in the band at 640 cm^–1^. These changes were noticeable, even at a low laser power density of 50 kW/cm^2^. (III) The formation of the C–C bonds occurred due to defects in the lattice structure. (IV) TiO_2_ structures started to form. It was noted that the reduction of surface groups was intertwined with the formation of C–C bonds and the beginning of the formation of TiO_2_ structures.

During the thermal treatment of MXene films, the most pronounced spectral changes occurred for samples heated to 200–300 °C. A red shift of the band at ∼620 cm^–1^ was observed as a sign of oxidation, but no blue shift of the band at ∼370 cm^–1^ was observed. However, a red shift of the latter band was observed for samples heated to 300 °C. It is important to note that in the study of laser-induced deterioration of MXene, the blue shift of the 370 cm^–1^ band was visible only until the formation of anatase began, after which a red shift was observed likewise. Based on the mode assignments of the homogeneous MXene, we can infer that the significant decrease in the bands at approximately 283, 435, and 620 cm^–1^ is associated with the Ti_3_C_2_(OH)_2_ MXene. Heating to 400 °C or above destroyed the MXene lattice. It should be noted that heating was conducted in a furnace and not under vacuum conditions. In the case of vacuum annealing, it is expected that carbon bands would still be visible at higher temperatures due to the absence of oxygen.

The differences in MXene degradation observed with laser and thermal treatments could be attributed to more severe temperature changes or different treatment conditions. In the furnace, the temperature is uniformly distributed across the entire sample. However, when heating with a laser, there is a significant temperature difference between the laser spot and the surrounding area. Another important difference to consider is the heating duration of the samples in the furnace, which was 30 min, while the laser treatment was limited to 5–10 min. As a result, the trends for disordered carbon and anatase formation were different.

The previously identified spectral changes induced by MXene lattice deterioration, caused by laser or thermal treatment, can be adapted to determine the MXene film aging. MXene films aged in oxygen and nitrogen environments differ from each other (Figure S3). The film kept in an oxygen environment is supposed to oxidize, increasing the number of =O surface functional groups. Indeed, the blue shift of the complex band at 372 cm^–1^ was only observed for MXene aged in an oxygen environment. However, the formation of TiO_2_ was not observed from the Raman spectra.

## Conclusions

This study focused on exploring lattice transitions in Ti_3_C_2_T_*x*_ MXene evoked by thermal and laser treatments using Raman spectroscopy as the primary analytical technique. In the laser-induced degradation study, the out-of-plane mode shifted with reduction in the interlayer water at lower power densities, followed by a blue shift of the band associated with the increasing =O content and diminishing of the bands of −OH-terminated MXenes. Amorphous carbon and hydrocarbons formed at excitation powers of 160 kW/cm^2^, while the formation of TiO_2_ nanoparticles (first the anatase phase, followed by a rutile phase) was observed at a power density of 550 kW/cm^2^ with 633 nm excitation wavelength. In the heating process, exposure to 100 °C led to a red shift of the out-of-plane mode, indicating the initiation of oxidation processes, while more pronounced oxidation and interlayer water reduction markers were observed at 200–300 °C. Finally, at 400 °C, a new band indicated the formation of anatase TiO_2_. Unlike laser treatment, no blue shift in certain bands was observed, indicating distinct degradation mechanisms under thermal conditions.

The findings presented here enhance the integration of Raman spectroscopy into MXene analysis by increasing our understanding of degradation pathways *via* related spectral markers. The critical optical and electric properties of MXene depend on structural changes, oxidation state, and the presence of amorphous carbon; thereby, the relation of Raman markers to degradation stages provides an invaluable tool for quickly assessing the structure of MXene. Further use of this knowledge enables the application of MXenes as components in more complex material structures while continuing to employ fast and straightforward Raman spectroscopy for characterization and analysis.

## Experimental Section

### Synthesis of Multilayered Ti_3_C_2_T_*x*_ MXene Colloidal Solutions

MXene used in this study was synthesized by selective etching Al layers from a Ti_3_AlC_2_ (Materials Research Center Ltd.; ≤40 μm particle size) MAX phase precursor. Detailed synthesis of multilayered Ti_3_C_2_T_*x*_ MXene proceeded as described elsewhere.^11^ Briefly, 0.1 g of Ti_3_AlC_2_ was gradually added to 5 wt % hydrofluoric acid (HF, Honeywell, 40 wt %, ACS, 7664-39-3) solution. The mixture was stirred at 200 rpm/min and kept on a thermostat for 24 h at 25 °C. After etching, the products were washed from the residues of acid with Milli-Q water by centrifugation in plastic centrifuge tubes at 2550 rcf for 10 min until the clear supernatant reached neutral pH. The sediments collected during centrifugation were multilayered Ti_3_C_2_T_*x*_ MXene.

### Synthesis of Single-Layered Ti_3_C_2_T_*x*_ MXene

To obtain single-layered MXene using the minimally intensive layer delamination (MILD) method,^60^ 0.1 g of Ti_3_AlC_2_ was gradually added to the 9 M hydrochloric acid (HCl, Roth, 37 wt %, ACS, 7647–01–0) solution with dissolved 1 g of LiF (Roth, >99%, 7789–24–4). The mixture was stirred at 200 rpm/min and kept on a thermostat for 48 h at 35 °C. The products were washed with Milli-Q water by centrifugation in plastic centrifuge tubes 3 times until the supernatant became dark, which indicates delamination. Then, centrifugation was continued, and a dark supernatant with delaminated, single-layered Ti_3_C_2_T_*x*_ MXene was collected. All substrates analyzed in this study were prepared by drop-casting of 0.01 g/mL solution of MXene on microscopic glass.

### Characterization of MXene Structures

Ti_3_C_2_T_*x*_ MXene morphology and elemental composition were evaluated by a scanning electron microscope Helios Nanolab 650 (FEI, Eindhoven, Netherlands) equipped with an EDX spectrometer X-Max (Oxford Instruments, Abingdon, U.K.). X-ray diffraction analysis was executed using Ni-filtered Cu Kα radiation on a MiniFlex II diffractometer (Rigaku, Tokyo, Japan) in Bragg–Brentano (θ/2θ) geometry within 2θ angle ranging from 5 to 60° with a step width of 0.02° and a sweep rate of 1°/min.

Optical characterization of MXene was performed using Vis–NIR and vibrational Raman spectroscopy. Extinction spectra were acquired in the spectral region 450–2300 nm using a Lambda 1050 UV–vis–NIR spectrophotometer (PerkinElmer). A MonoVista CRS+ Raman microscope (Spectroscopy & Imaging GmbH, Germany) equipped with 457, 532, 633, and 785 nm excitation lasers, 100×/0.8 NA objective, and a liquid nitrogen-cooled CCD detector was used for Raman analysis. All Raman spectra were collected using a 300 s exposure time. A 1500 lines/mm grating was employed to collect all spectra except those acquired with 785 nm excitation and for the 633 nm excitation, where a 300 lines/mm grating was used to cover the 100–1800 cm^–1^ spectral range. Before the measurements, the spectrometer was calibrated to the fundamental vibrational band of a silicon wafer at 520.7 cm^–1^.

For Raman spectroscopic analysis, the laser power was kept at ≈0.4 mW, which ensures a laser power density of ≈60 kW/cm^2^. The laser power was adjusted for the laser-induced deterioration study and is specified in the Results Section. The data in the deconvoluted Raman spectra and the low-frequency (100–800 cm^–1^) Raman spectrum of deteriorating Ti_3_C_2_T_*x*_ MXene in Figure 5 were smoothed using a Savitzky–Golay filter with a 9-point window. The unsmoothed spectra are provided in the Supporting Information.

For the evaluation of successful MXene synthesis, Raman mapping was conducted across a 10 × 10 μm^2^ area of the sample with a 1 μm step and no spectral markers of the MAX phase were observed. Additionally, both EDX and XRD analyses showed no trace of the MAX phase.

### Assessment of MXene Stability

The prepared MXene samples were subjected to different treatments: thermal annealing and storage under various conditions. For thermal annealing, the samples were placed in a furnace (Zhermack DM 40, Badia Polesine, Italy) by holding at 50, 100, 200, 300, 400, and 500 °C for 30 min. To study the stability of MXene under different storage conditions, MXene films were placed in a cuvette that was flushed and subsequently filled with dry oxygen or nitrogen gas and stored for 1 week at room temperature.

## Supporting Information Available

EDX mapping images of single-layered Ti_3_C_2_T*_x_* MXene flakes; vis–NIR extinction, Raman spectra and corresponding analysis of single-layered MXene flakes separately stored in oxygen and nitrogen gas environments; Raman spectra, analysis, and band assignment table of single-layered MXene obtained with different excitation wavelengths; Raman spectra of multilayered and single-layered MXene excited with 785 nm laser; Raman spectra of deteriorating Mxene due to laser illumination (457, 532, 633 nm) and due to heating (PDF)
