# Supplementary material for: Monitoring Ti3C2Tx MXene Degradation Pathways Using Raman Spectroscopy
Source: ACS Nano. 2024 May 6;18(20):13184–95. doi: 10.1021/acsnano.4c02150 (PMC11112979; doi:10.1021/acsnano.4c02150)
Supplement: Supplementary file 1 — nn4c02150_si_001.pdf [file nn4c02150_si_001.pdf]

Monitoring $Ti_3C_2T_x$ MXene Degradation Pathways Using Raman Spectroscopy

Sonata Adomavičiūtė-Grabusovė [1,*], Anton Popov [2], Simonas Ramanavičius [3], Valdas Šablinskas [1], Kateryna Shevchuk [4], Oleksiy Gogotsi [5] , Ivan Baginskiy [5,] Yury Gogotsi [4], and Arūnas Ramanavičius [6,7*]

1 Institute of Chemical Physics, Vilnius University, Sauletekio av. 3, LT-10257 Vilnius, Lithuania
2 NanoTechnas—Center of Nanotechnology and Materials Science, Faculty of Chemistry and Geosciences, Institute of Chemistry, Vilnius University, Naugarduko St. 24, LT-03225 Vilnius, Lithuania
3 Department of Organic Chemistry, Centre for Physical Sciences and Technology, Saulėtekio Av. 3, LT-10257 Vilnius, Lithuania
4 A.J. Drexel Nanomaterials Institute and Materials Science & Engineering Department, Drexel University, 3141 Chestnut Street, Philadelphia, PA 19104, U.S.A.
5 Materials Research Center, Ltd., Krzhyzhanovskogo str. 3, Kiev, Ukraine;
6 Department of Physical Chemistry, Faculty of Chemistry and Geosciences, Institute of Chemistry, Vilnius University, Naugarduko 24, LT-03225 Vilnius, Lithuania
7 Department of Nanotechnology, Centre for Physical Sciences and Technology, Saulėtekio Av. 3, LT-10257 Vilnius, Lithuania 5

* Correspondence: sonata.adomaviciute@ff.vu.lt (S.A.-G.); arunas.ramanavicius@chf.vu.lt (A.R.); simonas.ramanavicius@ftmc.lt (S.R.)

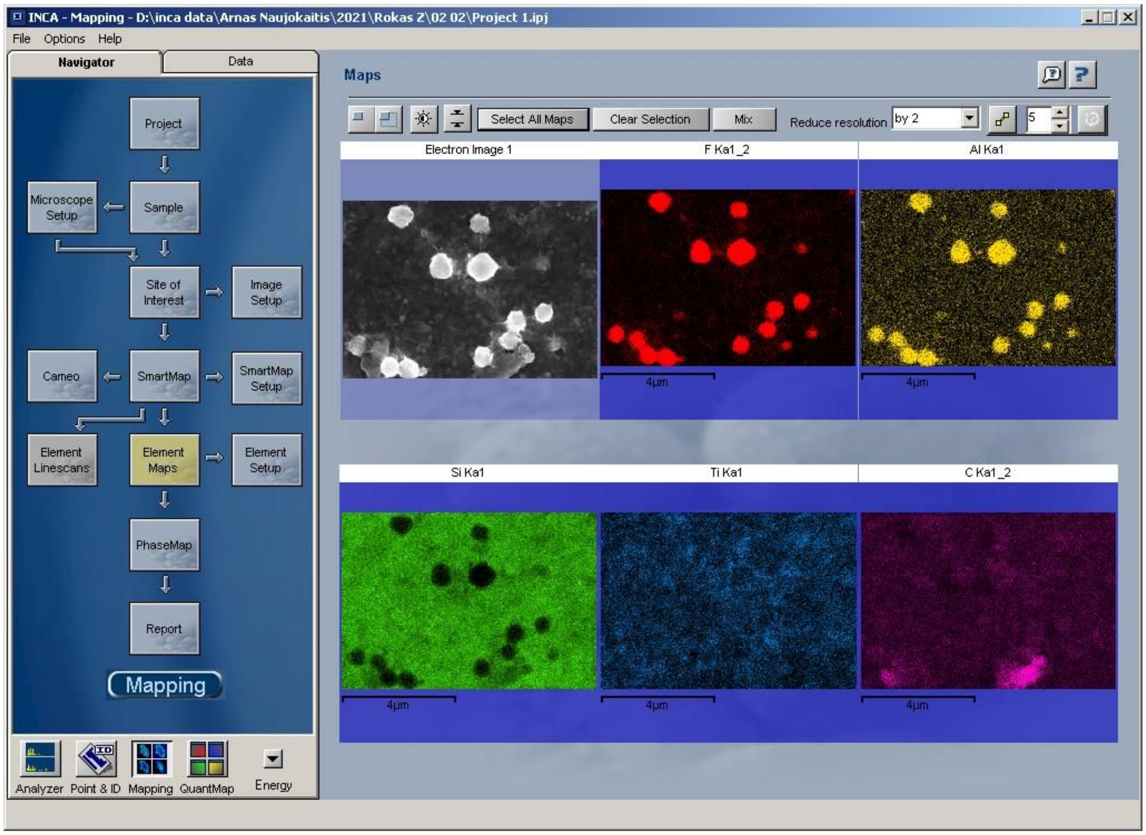

Figure S1. EDX mapping of exfoliated single-layered MXene flakes.

To study the stability of MXene, individual MXene films were stored for one week in a gas cuvette (1) with oxygen or (2) with nitrogen. The MXene film samples were then characterized by measuring Vis-NIR extinction and Raman spectra. The extinction spectra of the samples stored in different gas environments were compared with the extinction spectrum of MXene film annealed at 500 ºC.

For a comprehensive study of the quality of MXene flakes, MXene films were kept in an oxygen or nitrogen environment and investigated by Vis-NIR spectroscopy. The observed spectral differences between fresh and oxygenated films are thought to arise from changes in the surface groups with more $Ti_3C_2O_2$ and TiO_2 for the sample stored in an oxygen environment. The nitrogen environment was expected to slow down oxidation and lattice deterioration. In the spectrum of the oxygenated MXene film, a shift of the extinction band from ≈750 nm to ≈775 nm and an apparent decrease in optical density in the entire spectral range are observed. Recently, the increased conductivity due to the removal of MXene surface groups was reported.[1] The lower extinction of the oxidized MXene film may indicate more of =O surface groups. The film annealed at 500 °C (heated) reveals no extinction band since the organic material was incinerated, and only the TiO_2 remained on the sample, as evidenced by Raman spectroscopy performed later.

Vis-NIR extinction spectroscopy allows distinguishing between multilayered MXene and single-layered MXene flakes. In addition, the transmittance of MXene increased by 59% (from 11% to 27%) at 750 nm and by 72% (from 11% to 27%) at 2200 nm during oxidation. This suggests that Vis-NIR absorption spectroscopy seems to be a convenient and efficient method for determining the quality of the synthesized MXene.

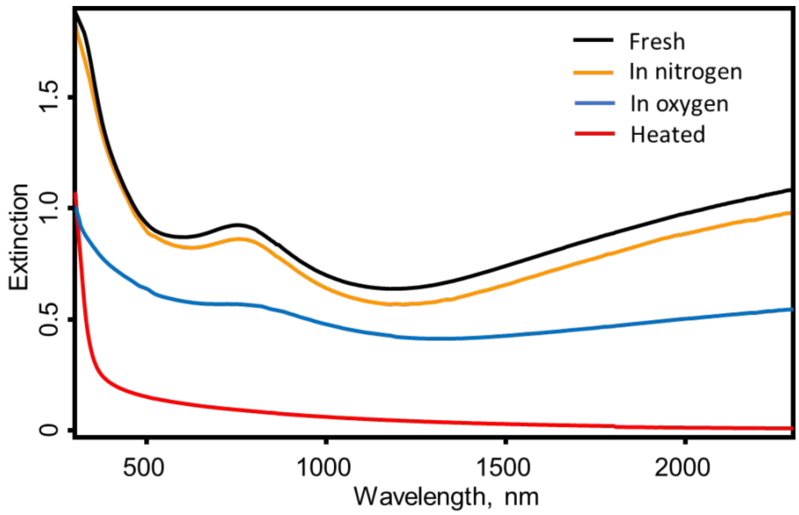

Figure S2. Vis-NIR extinction spectra of fresh MXene flakes (black line) and MXene flakes stored in cuvettes for one week with different gases: nitrogen (yellow line), oxygen (blue line), or annealed at 500°C (red line).

Raman spectroscopy can supply comprehensive information on the evolution of the MXene lattice structure. MXene films aged in oxygen and nitrogen environments differ from each other. It is assumed that the number of terminal groups that become oxygenated in the film aged in an

oxygen medium increases. The previously determined spectral changes in the MXene lattice can be adapted to MXene film aging in this case. Based on previously observed changes, one can see the recurring differences between the fresh MXene sample and MXene kept in different gas environments (Figure S3). A decrease in oxidation rate is observed for MXene kept in nitrogen. In contrast, the main differences are observed for the oxygenated MXene. Major markers include the ratios $I_{\omega2}/I_{\omega4(O)}$ and $I_{\omega4(O)}/I_{\omega4(OH)}$, which are 1.09 and 0.89 for the fresh sample, respectively. These ratios spike to 1.27 and 1 for the oxygenated sample. For sample kept in nitrogen environment these ratios are $I_{\omega2}/I_{\omega4(O)}$ - 1.16 and $I_{\omega4(O)}/I_{\omega4(OH)}$ - 0.9.

It is worth mentioning that the blueshift was observed for the band at 372 cm^{-1} and the redshift at 620 cm^{-1}. Such findings agree well with the results of other studies.[2–5] We can conclude that the formation of TiO_2 anatase has not yet advanced in this sample, so the blueshift at 372 cm^{-1} is still apparent. Furthermore, the terminal groups have been changed to =O. This can be traced from the $I_{\omega2}/I_{\omega4(O)}$ and $I_{\omega4(O)}/I_{\omega4(OH)}$ ratios.

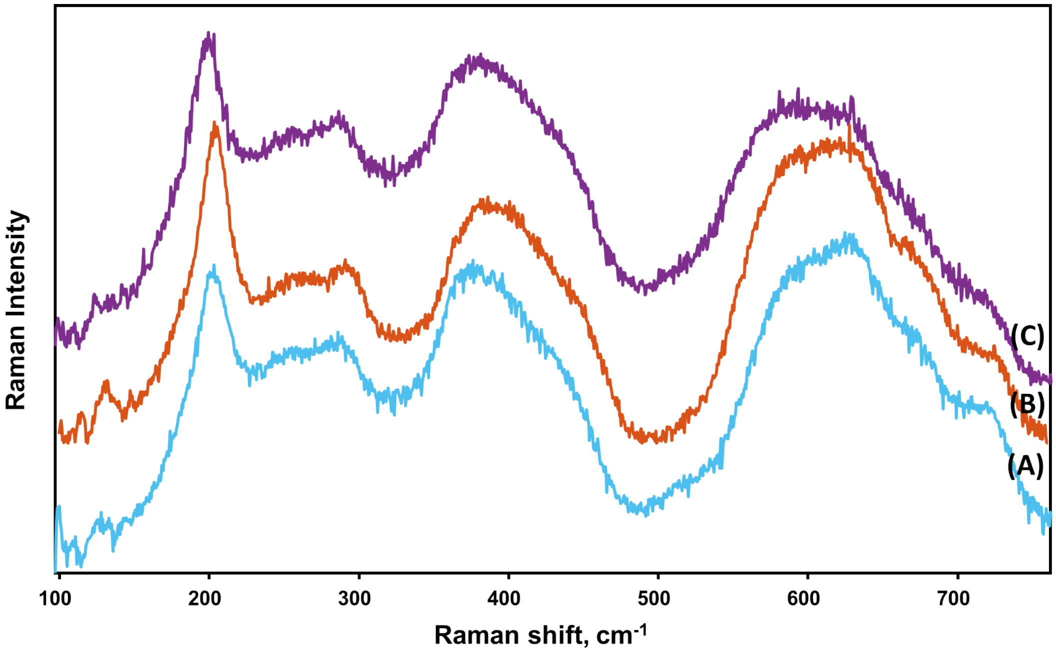

Figure S3. Raman spectrum of $Ti_3C_2T_x$ MXene in different environments. Fresh sample (A), after two weeks in nitrogen (B), and after two weeks in oxygen (C) (spectra are offset for clarity). The excitation wavelength was 633 nm.

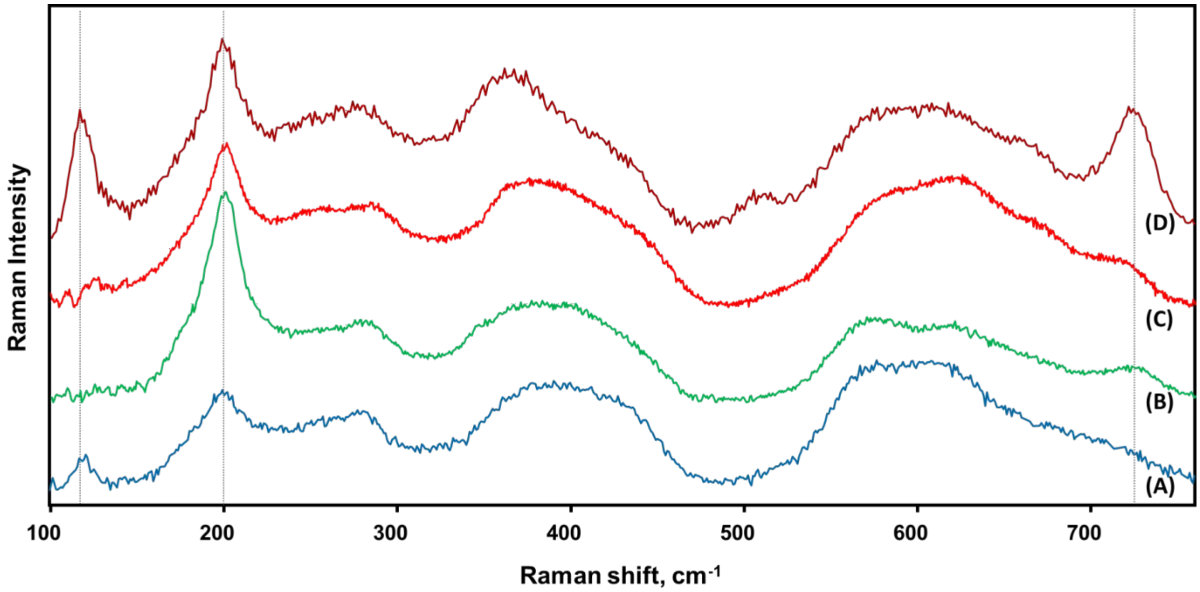

Figure S4. Raman spectra of single-layered $Ti_3C_2T_x$ MXene obtained with different excitation wavelengths: 457 nm (A), 532 nm (B), 633 nm (C), 785 nm (D).

When comparing the Raman spectra of single-layered MXene collected using different excitation wavelengths, significant changes were observed primarily under pre-resonance conditions (785 nm laser, as evident from Vis-NIR spectra). Additionally, pre-resonance conditions were observed for 457 nm (as evidenced by the increase in absorption towards the UV range in Vis-NIR spectra). These observations were reflected in the Raman spectrum, showing an increased resonance band at 120 cm^{-1} and shifts of the complex 370 and 620 cm^{-1} bands, which served as oxidation markers, as in our paper and the study by Berger et al.[6] Interestingly, the increased intensity in the 200 cm^{-1} band is observed for 532 nm excitation wavelength together with the redshift of the 620 cm^{-1} band. The minor differences between spectra can be attributed to variations in the MXene absorption capacity for other excitation wavelengths and differences in excitation power.

Excitation with a 785 nm laser provides photon energy close to the resonance Raman condition. The resonance Raman condition for MXene samples in our study was excitation with a 750 nm wavelength (Fig. 2). The pre-resonance excitation yields Raman spectra with a high intensity of several resonant spectral bands (Figure S4). These bands include 122 cm^{-1} (associated with the in-plane ω_1 vibrational mode of all atomic groups), 513 cm^{-1} (associated with the ω_6 mode of $Ti_3C_2(OH)_2$) and 722 cm^{-1} (associated mainly with the ω_3 mode of $Ti_3C_2O_2$) band. The complex band at approx. 372 cm^{-1} changes shape due to increased intensity in the spectral range at approx. 366 cm^{-1} and decreased intensity of the bands in the 380-460 cm^{-1} spectral range (compare with Figure S4 A and Figure S4 B). Overall, the most significant changes in intensity are observed in the spectral region for the surface groups. Similar spectral changes are observed for multi-layered MXene with pre-resonant (785 nm) and nonresonant (633 nm) excitations. The intensity of the resonant bands at 123 cm^{-1}, 512 cm^{-1}, and 737 cm^{-1} increases prominently. However, the spectral

range of the surface groups undergoes less enhancement (fewer surface groups overall), while the ω_2 mode undergoes relatively strong enhancement compared to single-layered MXene flakes.

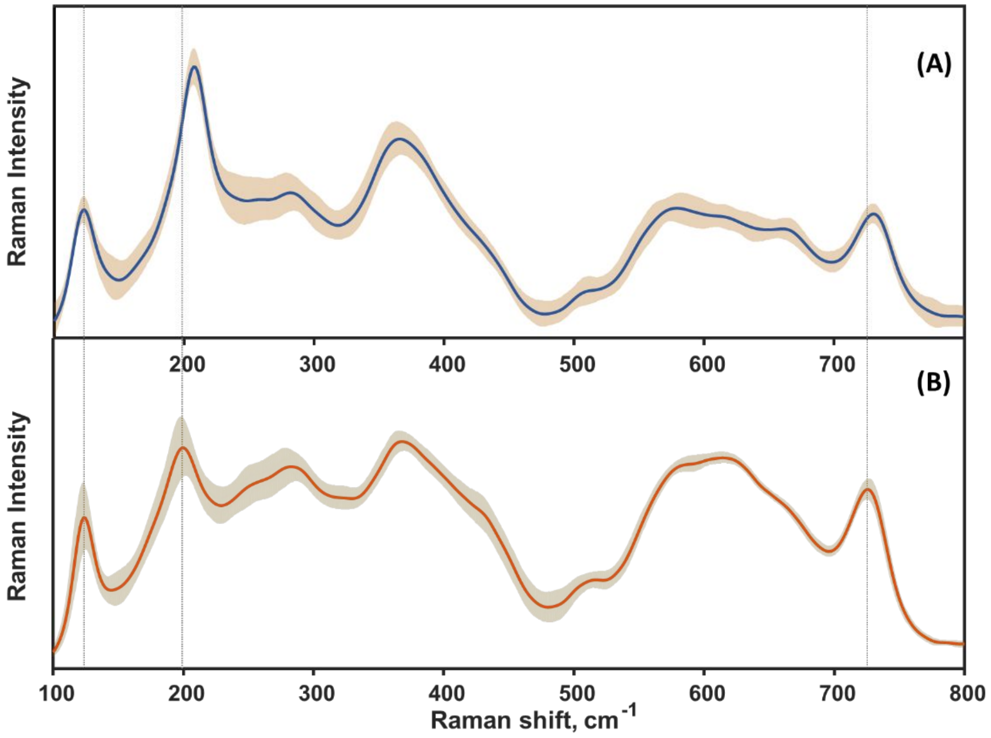

Figure S5. Raman spectra of multi-layered $Ti_3C_2T_x$ MXene (A) and $Ti_3C_2T_x$ MXene single-layer flakes (B). The excitation wavelength was 785 nm.

Table S1. Table of assignments of MXene spectral bands obtained with different excitation wavelengths: 457 nm (A), 532 nm (B), 633 nm (C) and 785 nm (D).

Vibrational frequency, cm^{-1}						Assignments[6,7]	MXene type
Single-layered flakes				Multilayered			
633 nm	785 nm	457 nm	532 nm	633 nm	785 nm		
122	122[a]	120	124	122	123[a]	$(Ti,F/O/OH,C)^{ip}$, ω_1	complex
154	154	154	155	154	154	Eg of TiO_2 ^{ip}	-
200	201	199	200	211	210	$(Ti,F/O/OH,C)^{oop}$, ω_2	complex
256	258	254	261	258w	258	$(F)^{ip}$, ω_5	$Ti_3C_2F_2$
283	283	278	283	284	283	$(OH)^{ip}$, ω_5	$Ti_3C_2(OH)_2$
372	372[a]	382	385	370	371[a]	ω_5	complex
450	-			452	-	$(OH)^{ip}$, ω_7	$Ti_3C_2(OH)_2$
511	513[a]	498	505	505w	512[a]	$(OH)^{oop}$, ω_6	$Ti_3C_2(OH)_2$
590	584	578	591	590	585	$(C)^{ip}$, ω_4	$Ti_3C_2O_2$
626	617	620	625	621	616	$(C)^{ip}$, ω_4	complex,
667	667	673	-	667	668	$\omega_3 + \omega_4$	$Ti_3C_2(OH)_2$
712	722[a]	708	720	734	737[a]	$(C)^{oop}$, ω_3	$Ti_3C_2O_2$
1396	1396			-	-	D band	
1582	1582		1575	1562	1561	G band	

[a] Increased intensity in spectral band compared to 633 nm excitation.
[W] Not prominent or weak band.
[ip] In-plane phonon mode.
[oop] Out-of-plane phonon mode.

Obtaining the Raman spectrum with infrared excitation (1064 nm) leads to strong absorption and heating of the sample. This behavior of MXene is of particular interest. Non-linear absorption was observed in $Ti_3C_2T_x$ MXene for this wavelength leading to saturable and enhanced optical absorbance. This behavior can be explained *via* plasmon-induced reinforcement of local electromagnetic fields, resulting in the enhancement of ground state absorption.[8–10]

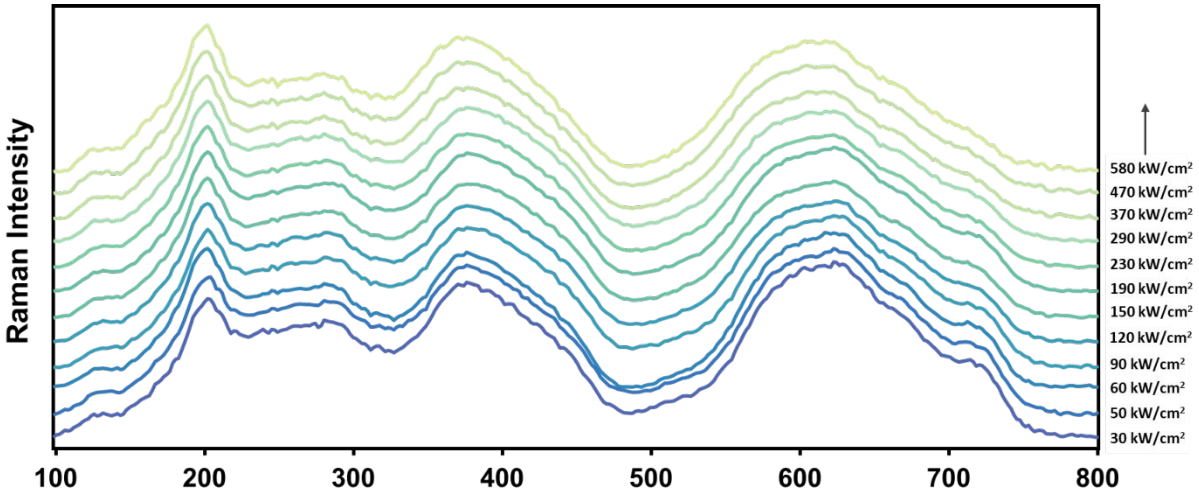

Figure S6. Raman spectrum of deteriorating $Ti_3C_2T_x$ MXene due to 633 nm laser illumination. The laser power density was varied from 30 to 580 kW/cm^2. The spectra are normalized to the spectral band at approximately 200 cm^{-1}.

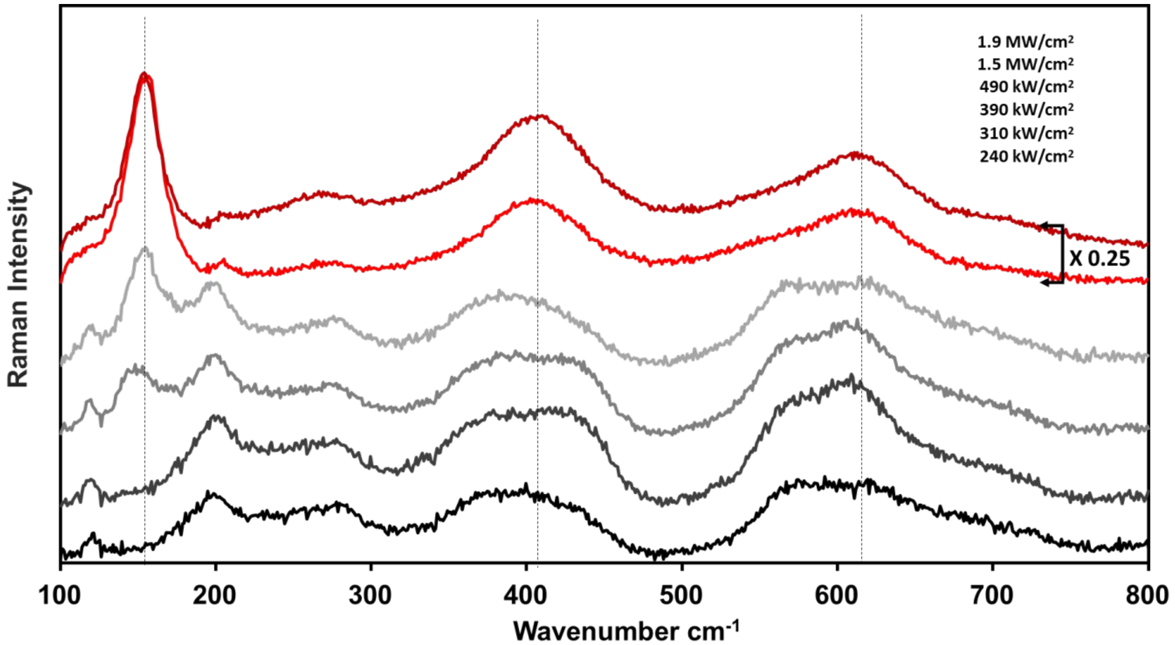

Figure S7. Raman spectrum of deteriorating $Ti_3C_2T_x$ MXene due to 457 nm laser illumination. The laser power density was varied from 240 kW/cm^2 to 1.9 MW/cm^2. The laser power density applied to the sample increases from bottom to top.

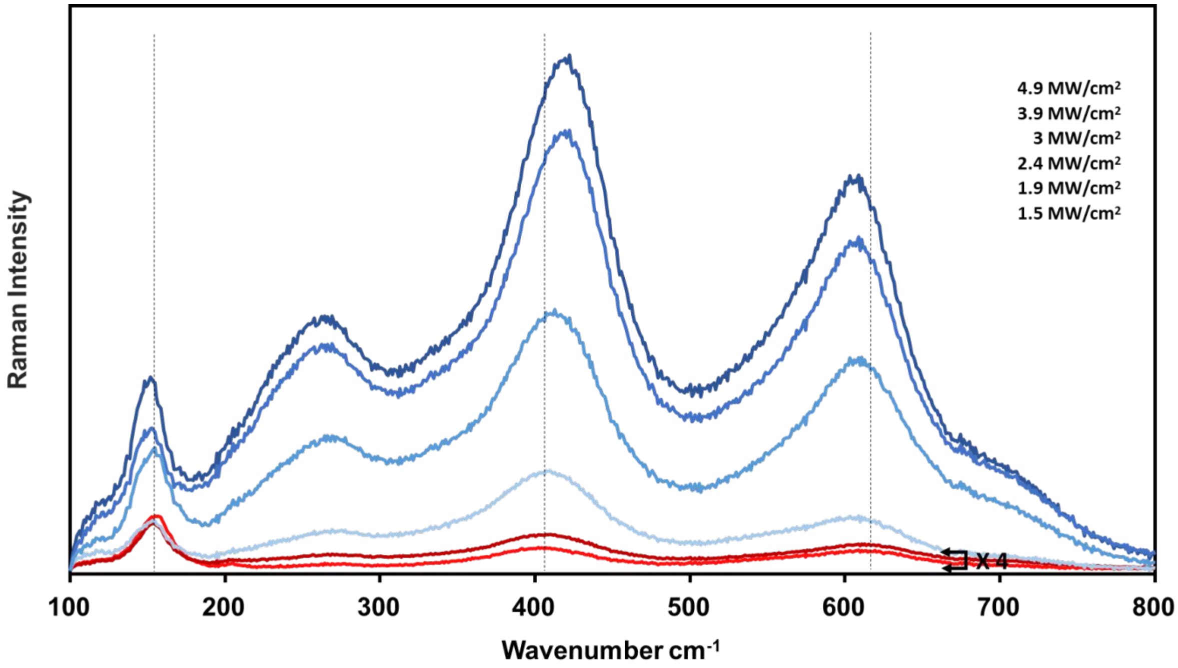

Figure S8. Raman spectrum of deteriorating $Ti_3C_2T_x$ MXene due to 457 nm laser illumination. The laser power density was varied from 1.5 to 4.9 MW/cm^2. The laser power density applied to the sample increases from bottom to top.

Similar changes in lattice deterioration are observed with 457 nm excitation. The anatase phase of TiO_2 appears from 310 – 390 kW/cm^2. A blueshift is observed for the complex band at approx. 380 cm^{-1} as a sign of MXene lattice oxidation, but it remains until the significant formation of TiO_2 is observable. The rutile phase becomes more prominent from 2.4 MW/cm^2. The evolution from the anatase phase to rutile is presented in Fig. S9, while the laser power density increases from 1.5 to 4.9 MW/cm^2.

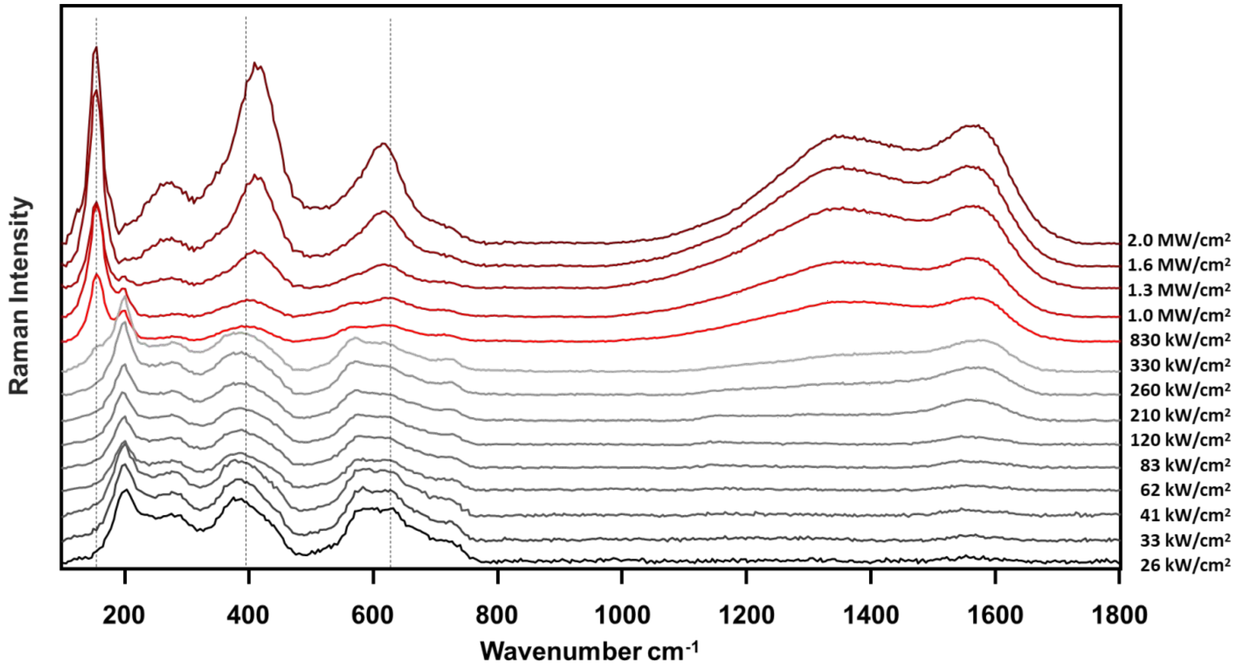

Figure S9. Raman spectrum of deteriorating $Ti_3C_2T_x$ MXene due to 532 nm laser illumination. The laser power density was varied from 26 kW/cm^2 to 2 MW/cm^2. The laser power density applied to the sample increases from bottom to top.

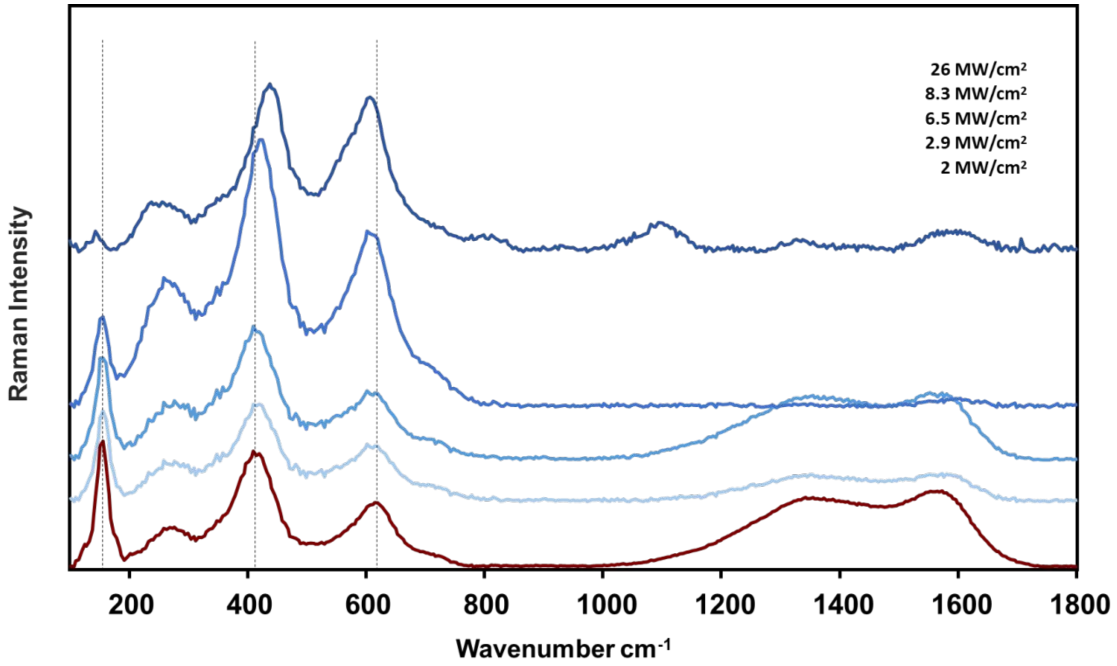

Figure S10. Raman spectrum of deteriorating $Ti_3C_2T_x$ MXene due to 532 nm laser illumination. The laser power density was varied from 2 MW/cm^2 to 26 MW/cm^2. The laser power density applied to the sample increases from bottom to top.

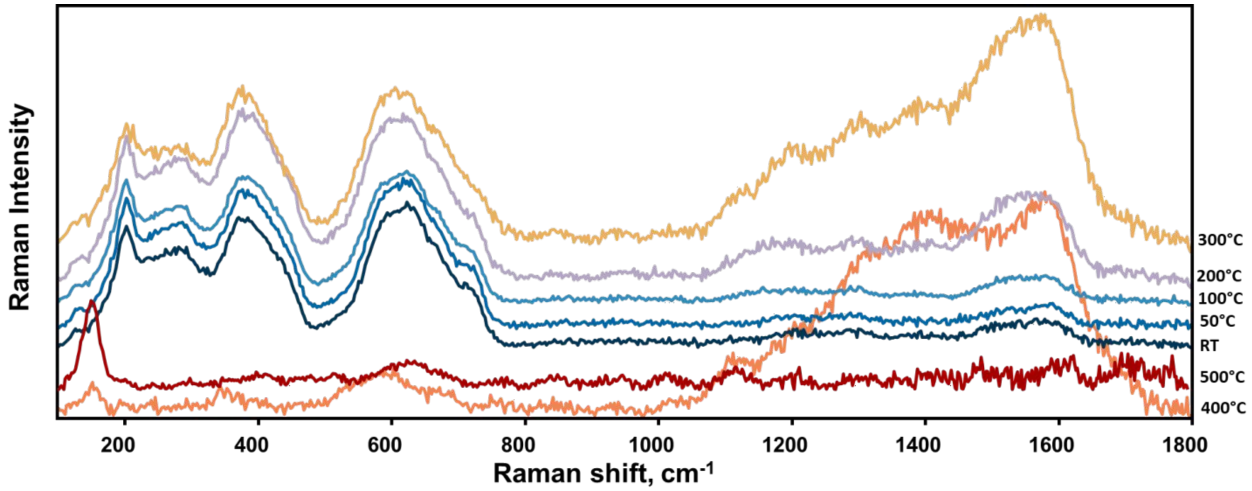

Figure S11. Raman spectra in 100-1800 cm^{-1} spectral region of $Ti_3C_2T_x$ MXene deteriorating due to heating. Raman excitation wavelength was 633 nm. MXene treatment temperatures: 50, 100, 200, 300, 400, and 500 °C. RT – room temperature.

References

(1) Shamsabadi, A. A.; Fang, H.; Zhang, D.; Thakur, A.; Chen, C. Y.; Zhang, A.; Wang, H.; Anasori, B.; Soroush, M.; Gogotsi, Y.; Fakhraai, Z. The Evolution of MXenes Conductivity and Optical Properties Upon Heating in Air. *Small Methods* **2023**, *7* (10), 2300568. https://doi.org/https://doi.org/10.1002/smtd.202300568.

(2) Li, H.; Chen, S.; Boukhvalov, D. W.; Yu, Z.; Humphrey, M. G.; Huang, Z.; Zhang, C. Switching the Nonlinear Optical Absorption of Titanium Carbide MXene by Modulation of the Surface Terminations. *ACS Nano* **2022**, *16* (1), 394–404. https://doi.org/10.1021/acsnano.1c07060.

(3) Benchakar, M.; Loupias, L.; Garnero, C.; Bilyk, T.; Morais, C.; Canaff, C.; Guignard, N.; Morisset, S.; Pazniak, H.; Hurand, S.; Chartier, P.; Pacaud, J.; Mauchamp, V.; Barsoum, M. W.; Habrioux, A.; Célérier, S. One MAX Phase, Different MXenes: A Guideline to Understand the Crucial Role of Etching Conditions on $Ti_3C_2T_x$ Surface Chemistry. *Appl Surf Sci* **2020**, *530*, 147209. https://doi.org/10.1016/J.APSUSC.2020.147209.

(4) Johnson, D.; Hansen, K.; Yoo, R.; Djire, A. Elucidating the Charge Storage Mechanism on Ti_3C_2 MXene through In Situ Raman Spectroelectrochemistry. *ChemElectroChem* **2022**, *9* (18), e202200555. https://doi.org/https://doi.org/10.1002/celc.202200555.

(5) Salles, P.; Pinto, D.; Hantanasirisakul, K.; Maleski, K.; Shuck, C. E.; Gogotsi, Y. Electrochromic Effect in Titanium Carbide MXene Thin Films Produced by Dip-Coating. *Adv Funct Mater* **2019**, *29* (17), 1809223. https://doi.org/https://doi.org/10.1002/adfm.201809223.

(6) Berger, E.; Lv, Z.-P.; Komsa, H.-P. Raman Spectra of 2D Titanium Carbide MXene from Machine-Learning Force Field Molecular Dynamics. *J Mater Chem C Mater* **2023**, *11* (4), 1311–1319. https://doi.org/10.1039/D2TC04374B.

(7) Hu, T.; Wang, J.; Zhang, H.; Li, Z.; Hu, M.; Wang, X. Vibrational Properties of Ti_3C_2 and $Ti_3C_2T_2$ (T = O, F, OH) Monosheets by First-Principles Calculations: A Comparative Study. *Phys Chem Chem Phys* **2015**, *17* (15), 9997–10003. https://doi.org/10.1039/C4CP05666C.

(8) Jhon, Y. I.; Koo, J.; Anasori, B.; Seo, M.; Lee, J. H.; Gogotsi, Y.; Jhon, Y. M. Metallic MXene Saturable Absorber for Femtosecond Mode-locked Lasers. *Adv Mater* **2017**, *29* (40), 1702496. https://doi.org/10.1002/adma.201702496.

(9) Jiang, X.; Liu, S.; Liang, W.; Luo, S.; He, Z.; Ge, Y.; Wang, H.; Cao, R.; Zhang, F.; Wen, Q. Broadband Nonlinear Photonics in Few-layer MXene $Ti_3C_2T_x$ (T= F, O, or OH). *Laser Photon Rev* **2018**, *12* (2), 1700229. https://doi.org/10.1002/lpor.201700229

(10) Song, Y.; Chen, Y.; Jiang, X.; Ge, Y.; Wang, Y.; You, K.; Wang, K.; Zheng, J.; Ji, J.; Zhang, Y. Nonlinear Few-layer MXene-assisted All-optical Wavelength Conversion at Telecommunication Band. *Adv Opt Mater* **2019**, *7* (18), 1801777. https://doi.org/10.1002/adom.201801777
